# Magnetosynthesis Effect on the Structure and Ground State of Cu^2+^-Based Antiferromagnets

**DOI:** 10.1021/acs.inorgchem.5c05555

**Published:** 2026-04-01

**Authors:** Micaela E. Primer, Anna A. Berseneva, Ayesha Ulde, Wenhao Sun, Rebecca W. Smaha

**Affiliations:** † Materials Science Center, 53405 National Laboratory of the Rockies, Golden, Colorado 80401, United States; ‡ Department of Materials Science and Engineering, 1259 University of Michigan, Ann Arbor, Michigan 48109, United States

## Abstract

Synthetic variables can have an outsized influence on the crystal structure and magnetic properties of a material, particularly those of quantum materials. In this work, we investigate the impact of synthesis under a magnetic field (magnetosynthesis) on the crystal structure and magnetic properties of several Cu^2+^ (*S* = 1/2)-based materials with antiferromagnetic interactions and varying levels of magnetic frustration, from simple antiferromagnets to a quantum spin liquid. Here, we develop methods to apply small (0.09–0.37 T) magnetic fields during low-temperature hydrothermal, evaporative, and rehydration syntheses of the simple antiferromagnet CuCl_2_·2H_2_O, the canted antiferromagnet (Cu,Zn)_3_Cl_4_(OH)_2_·2H_2_O, the frustrated and canted antiferromagnet atacamite Cu_2_(OH)_3_Cl, and the highly frustrated quantum spin liquid herbertsmithite Cu_3_Zn­(OH)_6_Cl_2_. We report the first single-crystal X-ray structural determination of the Cu_3_Cl_4_(OH)_2_·2H_2_O structure type and probe the stability of this phase both experimentally and computationally. Atacamite Cu_2_(OH)_3_Cl synthesized under a 0.19 T field experiences a 0.15 K (∼3%) decrease in its Néel transition temperature. This result suggests that magnetosynthesis with small applied fields may have a very subtle influence upon the magnetic properties of moderately magnetically frustrated 3*d* materials.

## Graphical abstract

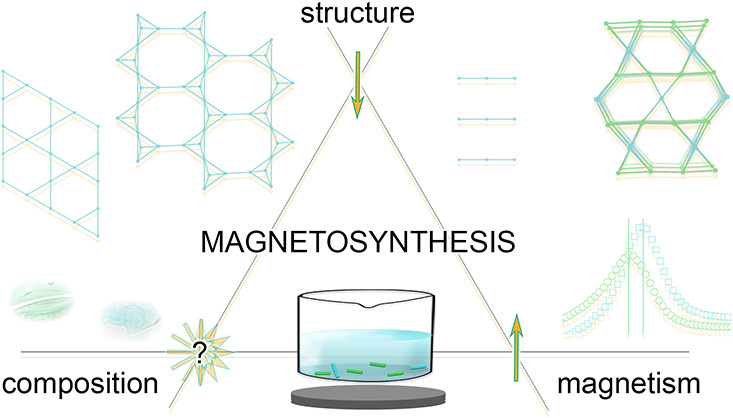

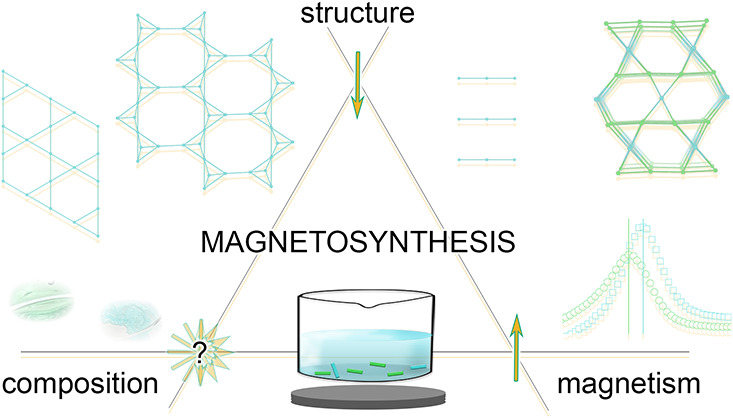

## Introduction

Frustrated antiferromagnets (AFMs) have several degenerate—or nearly degenerate—ground states and generally do not order until very low temperature (i.e., low Néel temperature *T*_N_) due to competing interactions caused by geometry, exchange interactions, or other factors. However, their Curie–Weiss temperature (Θ_CW_) is large and negative due to strong antiferromagnetic correlations. This juxtaposition led to the quantification of magnetic frustration as *f* = |Θ_CW_/*T*_N_|, where a large *f* value indicates a highly frustrated system.^1^ In the most extreme case where *T*_N_ = 0 and *f* = ∞, the material may be a quantum spin liquid (QSL). QSLs exhibit entanglement, a quantum mechanical correlation between electrons on different atoms. The long-range entanglement present in a QSL could aid in the development of quantum computers, and the spin liquid state could shield quantum qubits against outside noise.^2^

Slight changes in synthesis conditions can significantly influence the structure and spin states of materials, including quantum materials such as AFMs and QSLs. One reason for this may be that minor deviations in crystal structure affect the bond distances and exchange interactions between spins in magnetic structures. For example, small alterations in the synthesis recipe of the frustrated AFM barlowite Cu_4_(OH)_6_FBr, such as the precursor used, have been shown to affect its structure and magnetic properties.^3,4^ It is therefore important to investigate more broadly how various synthesis conditions can influence the crystal structures and spin state(s) of quantum materials and how they interact with a host of other factors such as composition, lattice, spin–orbit coupling (SOC) and J coupling, etc.

The most common synthetic variables include reagents, temperature, time, pH, and pressure. Magnetic field is a highly understudied synthetic parameter; most usage of a magnetic field has occurred as a postsynthetic treatment instead of during the initial synthesis. For example, application of a magnetic field during annealing is commonly performed to align ferromagnets and has also been known to change the properties of metals and alloys.^5,6^ Nonetheless, magnetosynthesis, the use of a magnetic field during synthesis, is an emerging field of research and has been shown to impact the crystal structure and magnetic properties of a few iridates and ruthenates.^7–9^

Cao et al. found that magnetosynthesis using only a 0.02–0.06 T field affected the magnetic properties and crystal structures of the AFM insulators Ba_4_Ir_3_O_10_, Sr_2_IrO_4_, and Ca_2_RuO_4_.^7^ As-synthesized Ba_4_Ir_3_O_10_ is a quantum liquid down to 0.2 K, yet magnetosynthesis induced long-range AFM order at the high temperature of *T*_N_ = 125 K and relieved the magnetic frustration.^7,9^ After magnetosynthesis at 0.02–0.06 T, the *T*_N_ of Sr_2_IrO_4_ was reduced from 240 to 150 K, and Ca_2_RuO_4_, which is normally an AFM with a *T*_N_ of 110 K, became ferromagnetic.^7,8^ Structural and electronic changes after magnetosynthesis were found as well, including reduced distortion in Ca_2_RuO_4_ and resistivity decreased by ∼5 orders of magnitude in Sr_2_IrO_4_. Note that these compounds all exhibit strong SOC due to their 4*d* and 5*d* metals, which Cao et al. hypothesized is key to the effectiveness of magnetosynthesis.

Here, we investigate the effect of magnetosynthesis upon the composition, crystal structure, and magnetic properties of a series of 3*d* Cu^2+^-containing materials exhibiting a range of magnetic frustration, from simple AFM (CuCl_2_·2H_2_O) to canted or frustrated/spin-glass AFMs ((Cu,Zn)_3_Cl_4_(OH)_2_·2H_2_O and atacamite Cu_2_(OH)_3_Cl, respectively) to a highly frustrated QSL material herbertsmithite (HBS, Cu_3_Zn­(OH)_6_Cl_2_). CuCl_2_·2H_2_O is a simple AFM with a low ordering temperature *T*_N_ = 4.3 K and a Θ_CW_ of around −5 K, so it is not frustrated.^10,11^ The compound Cu_3_Cl_4_(OH)_2_·2H_2_O has only been reported twice^12,13^ ; it is AFM with a *T*_N_ = 17.5 K and a Θ_CW_ of around +17.1, meaning it is also not magnetically frustrated.^13^ However, it exhibits a small net moment upon field cooling due to spin canting.^13^ Atacamite (Cu_2_(OH)_3_Cl) is a frustrated AFM (*f* ≈ 17) that exhibits spin glass behavior in synthetic samples.^14–19^ HBS (Cu_3_Zn­(OH)_6_Cl_2_) is the leading QSL candidate due to its arrangement of Cu^2+^ in an undistorted kagomé lattice.^20–22^

To target these phases, we develop various low-temperature magnetosynthesis methods that incorporate a permanent magnet: hydrothermal synthesis, evaporation of a salt solution formed during hydrothermal synthesis, and dehydration–rehydration. We report the first single crystal X-ray diffraction structural determination of the Cu_3_Cl_4_(OH)_2_·2H_2_O structure type. We also explore the stability and magnetosynthesis of mixed Cu/Zn versions of Cu_3_Cl_4_(OH)_2_·2H_2_O both experimentally and computationally; as Zn^2+^ is nonmagnetic, Zn^2+^ substitution onto Cu^2+^ sites is expected to influence its magnetic properties. This work explores the synthesis, stability, structure, and magnetic properties of four 3*d* Cu^2+^ compounds (CuCl_2_·2H_2_O, (Cu,Zn)_3_Cl_4_(OH)_2_·2H_2_O, atacamite Cu_2_(OH)_3_Cl, and herbertsmithite Cu_3_Zn­(OH)_6_Cl_2_) when synthesized with and without the presence of magnetic fields.

## Experimental Section

### Materials

Cu_2_(OH)_2_CO_3_ (54–56% Cu, Thermo Scientific), ZnCl_2_ anhydrous (99.95%, Alfa Aesar), CuO (+99%, ThermoFisher), ZnO (99.0%, Alfa Aesar), CuCl_2_·2H_2_O (reagent grade, Sigma-Aldrich), and HCl (ACS grade, Fisher Chemical).

### Measurement of Magnetic Fields

All magnetic fields were measured using a F.W. Bell model 5200 gaussmeter directly at the bottom interior of the vial, dish or autoclave (Scheme 1). There was often a range of magnetic fields found when the gaussmeter was applied in different locations on the bottom surface, as indicated by the errors within parentheses in the methods section. **Safety note: be careful to keep strong magnets separated from each other and other magnetic surfaces!**

**Scheme 1:**
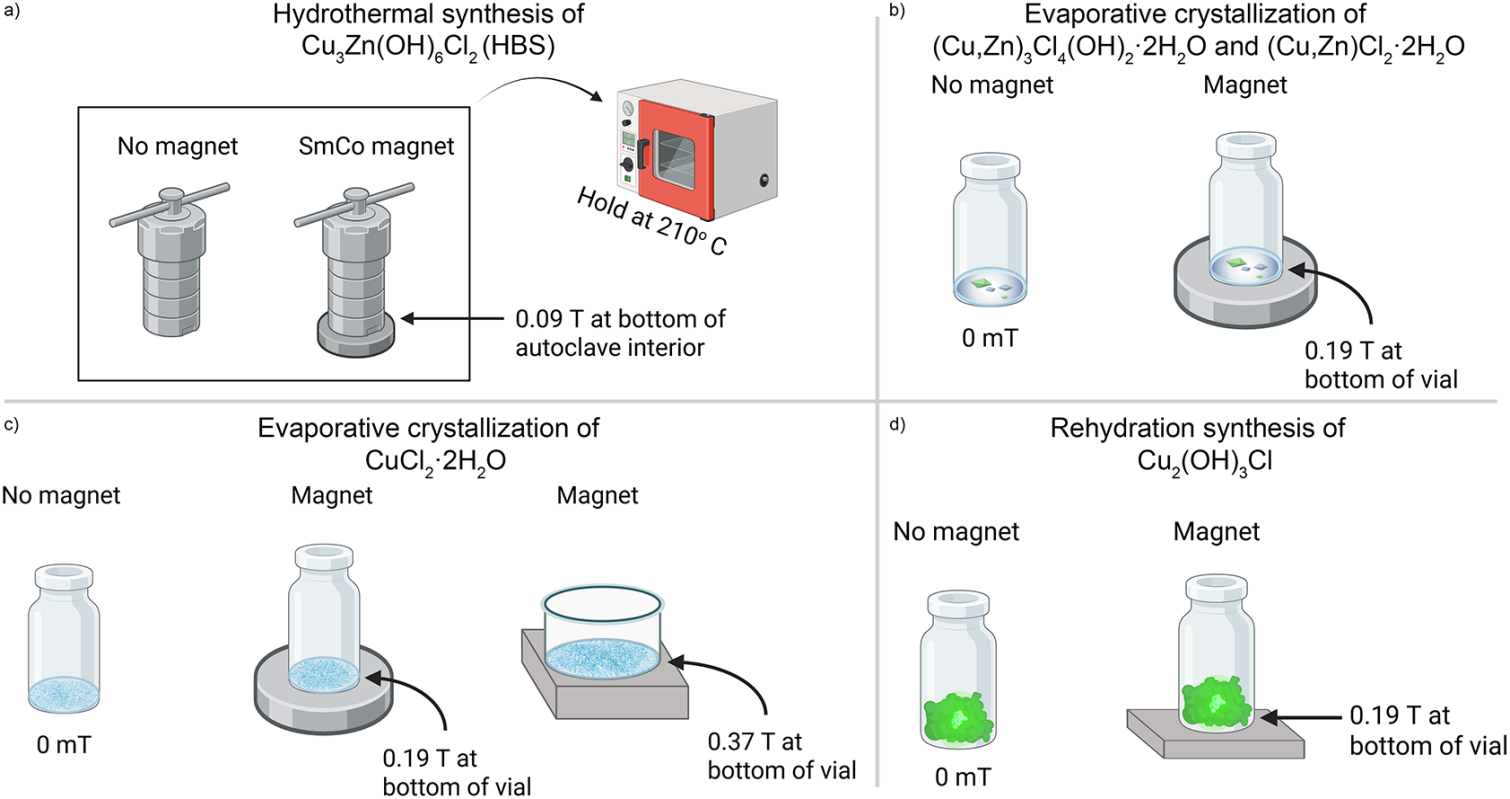
Synthesis Set-Ups for the Studied Materials: (a) HBS, (b) (Cu,Zn)_3_Cl_4_(OH)_2_·2H_2_O and (Cu,Zn)­Cl_2_·2H_2_O, (c) CuCl_2_·2H_2_O, and (d) Cu_2_(OH)_3_Cl^a^

a Figure created in BioRender. Primer, M. (2026) https://BioRender.com/x65d9am. Note: to enable comparison, syntheses with and without magnets were done in parallel. For all evaporative and rehydration attempts, samples were recrystallized from the same initial solution or powder, respectively, which was split between same-size vials; vials were left to evaporate/rehydrate in the same fume hood just far enough apart to not have a magnetic field propagate to the neighboring sample.

### Synthesis of HBS Cu_3_Zn­(OH)_6_Cl_2_

The reagents and stoichiometries shown in Table S1 were mixed and placed in hydrothermal autoclaves with 23 mL PTFE liners (Parr Instrument Company). The autoclaves were heated to 210 °C over 3 h, held at 210 *°*C for 24 h, and then cooled to room temperature over 30 h. The product was filtered and washed with acetone. One autoclave was placed above a heat-resistant samarium cobalt (SmCo) magnet that produced a magnetic field of 0.09 T at the bottom interior of the autoclave’s PTFE liner (Scheme 1a). The magnet was tested at 210 °C and retained its magnetism at this temperature. The other autoclave was heated without a magnetic field.

### Evaporative Crystallization of (Cu,Zn)_3_Cl_4_(OH)_2_·2H_2_O and CuCl_2_·2H_2_O

Three different trials were conducted, one with only Cu, one with only Zn, and one with a mixture of Cu and Zn. The reagents and stoichiometries shown in Table S2 were mixed and placed in hydrothermal autoclaves with 23 mL PTFE liners (Parr Instrument Company). The autoclaves were heated to 210 °C over 3 h, held at 210 *°*C for 24 h, and then cooled to room temperature over 30 h. Following the heating step, all solutions were placed in glass vials or Petri dishes and left to evaporate over the course of weeks. Some of the vials/dishes were placed above a magnet during evaporation. The Cu/Zn mixture evaporated in a vial with a magnetic field of 0.19(1) T at the bottom interior, and in a vial with no magnetic field (Scheme 1b). The pure Cu solution evaporated in vials with magnetic fields of 0 mT, 0.19(1) T, and 0.37(2) T at the bottom interior of the vials (Scheme 1c). The magnet that produced a field of 0.19(1) T was a SmCo magnet, and the magnet that produced a field of 0.37(2) T was an NdFeB magnet.

### Synthesis of Atacamite Cu_2_(OH)_3_Cl

The CuCl_2_·2H_2_O reagent was heated to 200 °C, held at 200 *°*C for 1 month, and then cooled to room temperature (Note that other attempts of this synthesis with shorter dwell times such as 2 weeks have also been promising). The resulting brown powder was split into two vials to rehydrate in a fume hood over the course of multiple weeks, until it became bright green. One vial was placed above a NdFeB magnet that produced a field of approximately 0.19(5) T at the bottom interior of the vial, and the other vial had no magnetic field during rehydration (Scheme 1d).

### Single-Crystal X-ray Diffraction (SCXRD)

We determined the crystal structures of the products with SCXRD using a Bruker D8 Venture with a Ga MetalJet source (1.341 Å) at 130 K and room temperature. The structures were solved with intrinsic phasing in APEX6 and refined with SHELXL and OLEX2.^23–27^ Hydrogen atoms were inserted at positions of electron density near the oxygen atoms and were refined with a fixed bond length and an isotropic thermal parameter 1.5 times that of the attached oxygen atom. Thermal parameters for all other atoms were refined anisotropically.

### Powder X-ray Diffraction (PXRD)

Products were ground using a mortar and pestle and placed on silicon zero-background slides with grease. PXRD measurements were taken using a Rigaku Ultima IV and Rigaku SmartLab in Bragg–Brentano geometry with Cu K_α_ radiation. HBS samples loaded in Kapton capillaries were also measured at Advanced Photon Source (APS) 17BM-B (λ = 0.25244 Å); images were calibrated with a LaB_6_ standard and integrated with GSAS-II.^28^ For the Rigaku Ultima IV, we used a 10 mm slit, a K_β_ filter, and a 0.02° step with a 0.5 h collection (or 8–12 h for scans used in the Rietveld refinement). For the Rigaku SmartLab, we used a 10 mm slit, a K_β_ filter, and a 0.001° step size with a 3.5 h collection. Rietveld refinements were performed using TOPAS and GSAS-II.^28,29^ Hydrogen atoms were excluded from the refinement model.

### Magnetism

AC susceptibility and DC magnetization measurements were performed using a Quantum Design Physical Properties Measurement System (PPMS) DynaCool. AC susceptibility was measured with a 5 Oe drive current at frequencies less than 1,500 Hz and with a 1.5 Oe drive current at frequencies between 1,500–10,000 Hz. DC magnetization measurements were performed under applied fields of −14 to 14 T and from 2 to 350 K, stabilizing at each temperature or applied field, respectively. For DC and AC measurements on atacamite samples, we measured the magnetic transition region with a 0.05 K step size; therefore, the error bar can be estimated as ±0.025 K. The magnetic field in the PPMS was nulled by oscillating it to zero well above the transition temperature before each measurement (except the field-cooled scans).

### Imaging and Composition Analysis

Scanning electron microscopy (SEM) imaging was done on a Hitachi S-4800 SEM operating at a 15 kV accelerating voltage and 10 μA beam current. Elemental analysis was conducted by energy-dispersive spectroscopy (EDS) on the same instrument using the included Pathfinder analysis software for quantification. Spectra were acquired for 60 s. EDS mapping was performed by acquiring data for 6 min.

### Wavelength Dispersive X-ray Fluorescence (WD-XRF)

Wavelength dispersive X-ray fluorescence (WD-XRF) was performed to semiquantitatively assess composition using a Rigaku ZSX PrimusIV. Data collection was performed on powder dispersed on Kapton tape. Note that O element is always present in this setup coming from the Kapton tape and therefore was not quantified.

### First-Principles Calculations

Spin-polarized (collinear) density functional theory (DFT) calculations were conducted to compute the quantum mechanical energies for the 2 × 2 × 2 supercell of (Cu,Zn)_3_Cl_4_(OH)_2_·2H_2_O using the Vienna Ab-Initio Package (VASP),^30,31^ with the Projector Augmented-Wave method^32^ using the Perdew–Burke–Ernzerhof (PBE) generalized gradient approximation.^33^ DFT basis cutoff energies were 520 eV and the Gamma point was used for the k-point mesh. (Cu,Zn)_3_Cl_4_(OH)_2_·2H_2_O exhibits canted AFM, but in the absence of experimental information on its magnetic propagation vector, all calculations were performed assuming a collinear ferromagnetic ordering. The formation energies were referenced to the elemental DFT free energies provided by the Materials Project^34^ and adjusted using the MaterialsProject2020Compatibility scheme^35^ implemented in Pymatgen.^36^

## Results

### Magnetosynthesis of HBS

HBS Cu_3_Zn­(OH)_6_Cl_2_ is the leading experimental kagome QSL candidate material,^20–22^ so we initially focused our hydrothermal synthesis and magnetosynthesis attempts on this phase. However, it can be challenging to synthesize pure HBS due to the tendency of Cu to substitute on the interlayer Zn sites, and even the purest phases tend to have ∼15% Cu on the Zn sites (i.e., Cu_3_Zn_0.85_Cu_0.15_(OH)_6_Cl_2_).^37,38^ As a starting point, we used Cu_2_(OH)_2_CO_3_ and anhydrous ZnCl_2_ (see Table S1 for details) mixed with water and heated in a PTFE-lined autoclave at 210 °C for 24 h. A large excess of ZnCl_2_ was required to eliminate tenorite (CuO) impurities identified via PXRD (Figure S1), consistent with prior attempts.^39^

The optimized synthesis was repeated both with and without a SmCo permanent magnet under the autoclave, which yielded a magnetic field of 0.09 T at the bottom of the PTFE liner (Scheme 1a). Throughout the manuscript, we label samples with the field used to synthesize them as, e.g., HBS_0T and HBS_0.09T. Both products were pure according to PXRD and visual inspection (Figure 1a). We performed Rietveld fits of the PXRD data for each sample, and the extracted lattice parameters were identical within error (Figure S8, Tables S4, and S5). Wavelength dispersive X-ray fluorescence (WD-XRF) on the HBS powders resulted in similar formulas Cu_3_Zn_0.81_Cu_0.19_(OH)_6_Cl_2_ and Cu_3_Zn_0.79_Cu_0.21_(OH)_6_Cl_2_ for HBS_0T and HBS_0.09T samples, respectively.

**Figure 1:**
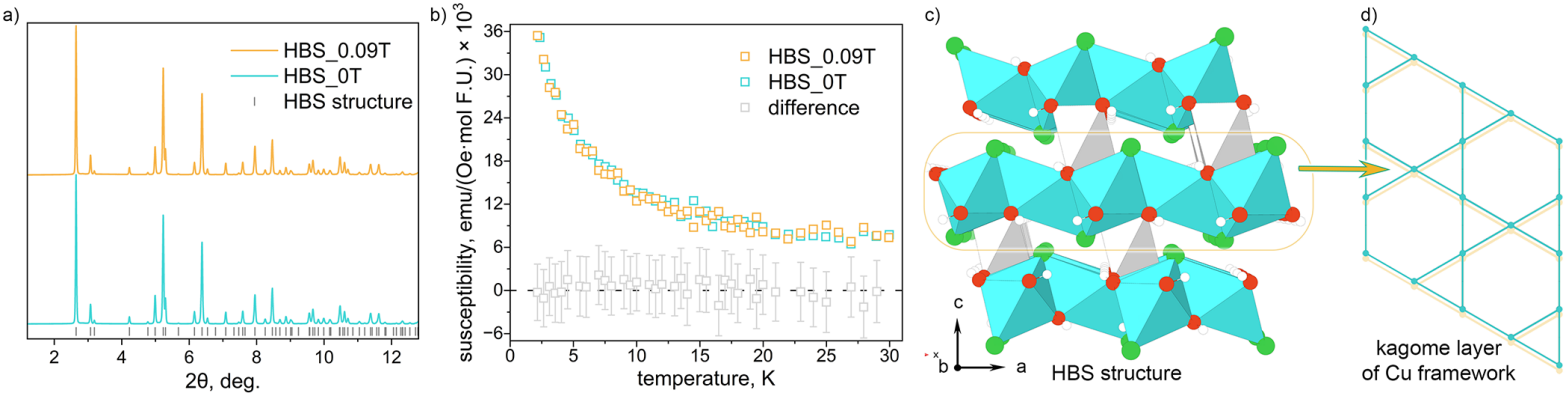
(a) PXRD data comparing the HBS synthesis under 0.09 and 0 T. (b) ZFC magnetic susceptibility (measured at 0.005 T) of HBS synthesized with and without 0.09 T magnetic field as well as the difference between them. (c) View of HBS structure and (d) kagome layer built from the Cu-framework. White, red, green, blue, and gray spheres, and blue and gray polyhedra represent H, O, Cl, Cu, and Zn atoms and Cu and Zn octahedra, respectively.

The low temperature magnetic susceptibility data of HBS synthesized under a 0.09 T magnetic field were indistinguishable from that of HBS synthesized with no field (Figure 1b). The data for both samples are consistent with QSL behavior: there is no magnetic transition down to 2 K. For both 0 and 0.09 T syntheses, the zero field-cooled (ZFC) and field-cooled (FC) susceptibilities were identical (Figures S18 and S19), matching the results found by Shores et al. for HBS synthesized without a magnetic field.^20^ Moreover, Curie–Weiss fits in the 100–350 K temperature range resulted in similar Curie–Weiss temperatures (Θ_CW_) and effective moments (μ_eff_) for Cu^2+^ (Table S17 and Figure S20), corroborating that 0.09 T does not have a strong effect on the synthesis of HBS.

### Crystal Growth of Metastable Cu-Containing Chlorides

Next, we explored the synthesis of HBS using CuO and ZnCl_2_ reagents to grow single crystals and potentially increase the yield due to impact on the CuO vs Cu_2_(OH)_2_CO_3_ dissolution kinetics in hydrothermal conditions. However, it was more difficult to produce a pure product using CuO than Cu_2_(OH)_2_CO_3_: at a 0.08:1 Cu:Zn ratio, the product was still contaminated with CuO impurities, and only extreme ratios of 0.04:1 produced small amounts of pure product at 5% yield (Table S1). Therefore, we added concentrated HCl to the hydrothermal reactions. At a high enough concentration, HCl successfully dissolved CuO; however, the HBS powder also dissolved, and no product precipitated. We then used these clear solutions for evaporative crystallization under no and small magnetic fields at room temperature to grow single crystals of Zn/Cu/Cl-containing materials (Scheme 1b). To compare the effect(s) of Cu^2+^ and Zn^2+^ ions on crystallization dynamics, we prepared aqueous solutions of Cu and/or Zn chlorides with HCl (see Table S2 for details) and heated them in a PTFE-lined autoclave at 210 °C for 24 h. Solutions were prepared with either only Cu, only Zn, or mixed Cu/Zn chlorides. The solutions were then permitted to evaporate at room temperature in Petri dishes.

Immediately after the heating step, the Zn-only solution was clear. Upon evaporation for 3–5 weeks, a clear liquid layer remained; no crystals formed (Figure 2). However, the Cu-only solution was dark green after the heating step. After a few days, the liquid evaporated fully and loose clusters of light blue flaky crystals remained. Unfortunately, these crystals were not suitable for SCXRD; therefore, this phase was identified as CuCl_2_·2H_2_O by PXRD.

**Figure 2:**
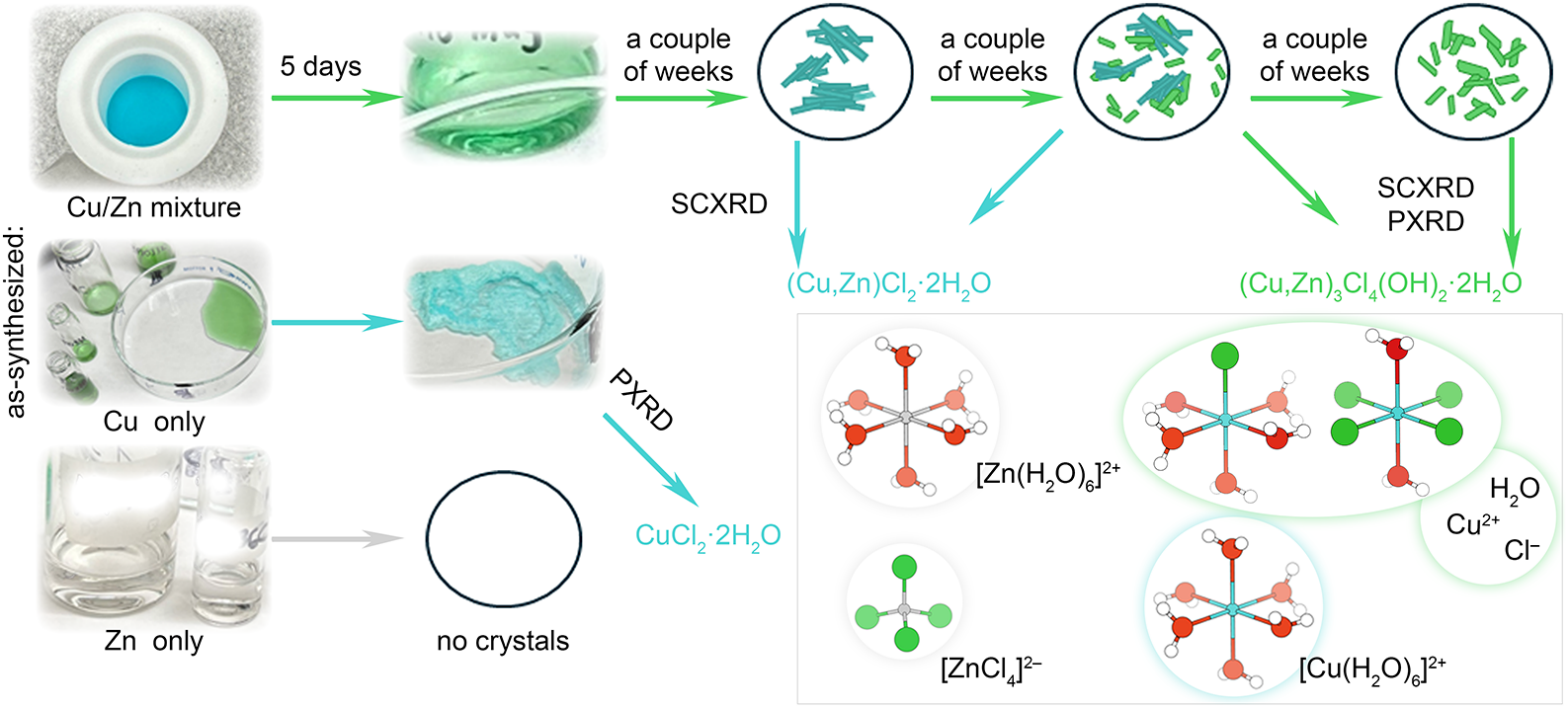
Evaporative crystallization from Cu and/or Zn solutions after hydrothermal reaction. The bottom right inset shows the main complexes present in the solution after hydrothermal reaction. White, red, green, blue, and gray spheres represent H, O, Cl, Cu, and Zn atoms, respectively. The color-coding of the complexes corresponds to their appearance in solution.

The most interesting case was presented by the mixed Cu/Zn solution: after heating, it was a bright/light blue color. After a few days, the solution became teal green/blue (Figure 2). Once the liquid was almost evaporated, blue crystals formed. Several weeks later, as the liquid continued to evaporate, blue and green crystals were both present. Interestingly, we could see the intergrowth between green and blue crystals in an optical microscope. Yet a couple weeks after that, only green crystals remained. We note that the liquid never fully evaporated; a mother liquor was retained surrounding the crystals. The blue crystals were long needles, sometimes even reaching a few millimeters in length. The green crystals were smaller and exhibited an irregular hexagonal plate-like morphology. Through SCXRD, the blue crystals were identified as (Cu,Zn)­Cl_2_·2H_2_O (isostructural to CuCl_2_·2H_2_O, as suggested by a unit cell check), and the green crystal structure was solved as (Cu,Zn)_3_Cl_4_(OH)_2_·2H_2_O (isostructural to Cu_3_Cl_4_(OH)_2_·2H_2_O in space group *P*–1, Tables S6–S10); this structure is described in more detail below.

Despite the similarity of Cu^2+^ and Zn^2+^ in terms of oxidation state and ionic radius (0.73 Å and 0.74 Å for Cu^2+^ and Zn^2+^ in octahedral coordination, respectively^40^), the results of these evaporations varied widely. One major difference between Cu^2+^ (*d* ^9^) and Zn^2+^ (*d* ^10^) is that Cu^2+^ experiences Jahn–Teller effects due to its unpaired electron, whereas Zn^2+^ does not. We speculate that these effects lead to the instability or metastability of the blue (Cu,Zn)­Cl_2_·2H_2_O phase—it slowly transforms to the green (Cu,Zn)_3_Cl_4_(OH)_2_·2H_2_O phase, which thus is more thermodynamically stable. SEM images of the blue and green crystals show that the blue crystals are more granular than the green, making (Cu,Zn)­Cl_2_·2H_2_O easier to redissolve and convert to (Cu,Zn)_3_Cl_4_(OH)_2_·2H_2_O (Figure S3). We hypothesize this is likely because the lattice does not easily accommodate Zn^2+^ in the irregular octahedral sites. Additionally, upon tallying known compounds involving Zn and Cu, Zn^2+^ coordinated to Cl^–^ tends to prefer a tetrahedral geometry, especially if the majority of ligands are Cl^–^, whereas Cu^2+^ coordinated to Cl^–^ prefers octahedral or square pyramidal geometries (Table S3). In CuCl_2_·2H_2_O, Cu^2+^ is coordinated in an octahedral geometry to four Cl^–^ and two H_2_O, which may contribute to the lack of stability of Zn^2+^ in these sites (see Figure 3a). Apart from that, the differences and progression in the solution color provides information about Cu^2+^ coordination environment in aqueous media. For the Cu^2+^-only solution, after hydrothermal dissolution of CuO in HCl the solution is green, indicating the presence of complexes such as [Cu­(H_2_O)_5_Cl] ^2+^ and [Cu­(H_2_O)_2_Cl_4_] ^2+^ (Figure 2).^41^ After evaporative crystallization, this solution turned blue, corroborating the evaporation of residual HCl and Cl^–^ being incorporated into the CuCl_2_·2H_2_O structure, hence leaving the blue colored [Cu­(H_2_O)]_6_^2+^ ions in solution (Figure 2).^41^ Opposite to that, hydrothermal dissolution of CuO and ZnCl_2_ leads to [Cu­(H_2_O)]_6_^2+^ species in the aqueous media resulting in blue color potentially due to preferential Zn^2+^ bonding to Cl^–^. This solution later turns green; therefore, it is likely to contain [Cu­(H_2_O)_5_Cl]^2+^ and [Cu­(H_2_O)_2_Cl_4_]^2+^, since the presence of Zn^2+^ might preserve more Cl^–^ ions in solution and let H_2_O evaporate slower, producing better quality crystals (Figures 2, S4, and S5).

**Figure 3:**
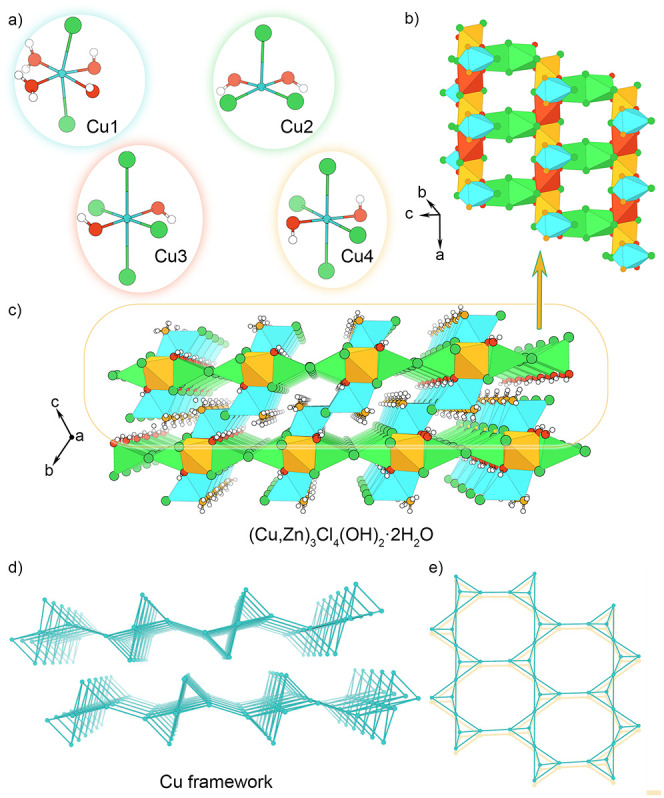
View of (a) building polyhedral units, (b) (Cu,Zn)_3_Cl_4_(OH)_2_·2H_2_O layers, (c) overall (Cu,Zn)_3_Cl_4_(OH)_2_·2H_2_O structure, and (d, e) Cu framework. White, red, green, blue, and orange spheres represent H, O, Cl, and Cu atoms and water molecules, respectively. Blue, green, red, and orange polyhedra represent Cu1, Cu2, Cu3, and Cu4, respectively. Zn atoms in the structure are omitted for clarity.

### Crystal Structures of (Cu,Zn)_3_Cl_4_(OH)_2_·2H_2_O Obtained under No and Weak Magnetic Field

The Cu_3_Cl_4_(OH)_2_·2H_2_O structure has only two references in the literature.^12,13^ One of the previous synthetic approaches involved the solid state reaction between CuCl_2_·2H_2_O and Cu­(OH)_2_ powders in air at 75 °C for 5 days followed by washing with alcohol and drying at 75 °C.^12^ Another route consisted of a nontrivial cycle: dehydration of CuCl_2_·2H_2_O at 200 °C for several weeks, rehydration at room temperature, and wetting the resulting powder with a couple of water drops followed by drying at 50–60 °C.^13^ In contrast to our (Cu,Zn)_3_Cl_4_(OH)_2_·2H_2_O crystal growth method, only powders of Cu_3_Cl_4_(OH)_2_·2H_2_O were produced by Walter-Lévy et al. in 1970 and Asaf et al. in 1996.^12,13^ The authors also mentioned that it was challenging to obtain crystalline and phase-pure material.^13^

Here, we took advantage of the room-temperature evaporative crystal growth of (Cu,Zn)_3_Cl_4_(OH)_2_·2H_2_O to synthesize single crystals and probe the effect of a 0.19 T magnetic field upon the resulting material (Scheme 1b). In both synthetic routes, we obtained green block-shaped crystals after a couple of weeks (depending on temperature, humidity, airflow, etc.) and employed SCXRD to determine that the crystals were (Cu,Zn)_3_Cl_4_(OH)_2_·2H_2_O (sp. gr. *P*–1). This phase is isostructural to Cu_3_Cl_4_(OH)_2_·2H_2_O, which had previously only been characterized by PXRD.^12,13^ We therefore present here the first full structure determination of this type, which is a layered structure accommodating four different Cu^2+^/Zn^2+^ sites coordinated by Cl^–^, OH^–^, and H_2_O ligands. As shown in Figure 3a, Cu1, Cu3, and Cu4 are in distorted octahedral geometries, while Cu2 is in distorted square pyramidal geometry. The layers in (Cu,Zn)_3_Cl_4_(OH)_2_·2H_2_O (Figure 3b,c) are built by chains of Cl,Cl-edge sharing [CuCl_4_(OH)_2_] octahedra with Cu3 and Cu4 alternating within the 1D moiety. These chains are connected by Cl,Cl-edge sharing Cu2 dimers, i.e., [CuCl_3_(OH)_2_]_2_, via Cl,OH-edge sharing to both Cu3 and Cu4 octahedra to create a rectangular mesh (Figure 3b). And finally, this sheet is decorated by terminal Cu1 octahedra, [CuCl_2_(OH)_2_(H_2_O)_2_], through OH,OH-edge sharing with the Cu2-containing square pyramid and Cl,OH-edge sharing with Cu3 and Cu4 octahedra (Figure 3b).

Even though the (Cu,Zn)_3_Cl_4_(OH)_2_·2H_2_O layers are charge-neutral, there is H···O and H···Cl hydrogen bonding leading to the layer stacking in the (Cu,Zn)_3_Cl_4_(OH)_2_·2H_2_O structure (Figure S10). Moreover, the terminal Cl1 ligand coordinated to Cu1 is significantly further from Cu2 in the adjacent layer (3.229 Å at 300 K), having a weak ionic interaction further gluing the layers together (Figure S11). However, this layered structure did not demonstrate signs of exfoliation as shown in the SEM images (Figures S3 and S4), and it does not easily dissolve in water, isopropanol, or acetone primarily due to the strong hydrogen bonding and weak ionic connection between layers.

We note that SCXRD cannot conclusively distinguish Cu^2+^ and Zn^2+^ due to their nearly identical *Z* and ionic radii. However, we attempted to refine different occupancy models against the SCXRD data, which resulted in empirical formulas of Cu_2.86_Zn_0.14_Cl_4_(OH)_2_·2H_2_O (RT, 0 T), Cu_2.87_Zn_0.13_Cl_4_(OH)_2_·2H_2_O (130 K, 0 T), and Cu_2.93_Zn_0.07_Cl_4_(OH)_2_·2H_2_O (130 K, 0.19 T), although the R-values were not significantly improved. The EDS results for (Cu,Zn)_3_Cl_4_(OH)_2_·2H_2_O crystal synthesized without an applied field suggested a Cu:Zn:Cl ratio of approximately 2.85:0.15:5.01 (Figure S5), and we thus used this Cu:Zn ratio to finalize the (Cu,Zn)_3_Cl_4_(OH)_2_·2H_2_O crystal structures.

Interestingly, the only location for Zn^2+^ to substitute that did not result in refinement instability or negative occupancies was on the Cu1 site. Our hypothesis is that this position is more stable because only two Cl^–^ are bonded to Cu1 in this octahedral coordination environment, as opposed to four, and Cl^–^ ligands with Zn^2+^ would usually prefer to be tetrahedral but substitution of Cl^–^ to H_2_O or OH^–^ shifts Zn^2+^’s preferred geometry to octahedral coordination (Table S3). We therefore posit that Zn^2+^ substitution destabilizes CuCl_2_·2H_2_O, in which the metal site has octahedral coordination, and promotes formation of the Cu_3_Cl_4_(OH)_2_·2H_2_O structure with diverse octahedral metal sites. These hypotheses explain why the (Cu,Zn)_3_Cl_4_(OH)_2_·2H_2_O crystal is more thermodynamically stable in the mixed Cu/Zn trial.

To probe our hypothesis that Zn substitution stabilizes the (Cu,Zn)_3_Cl_4_(OH)_2_·2H_2_O structure, we performed spin-polarized DFT calculations on the 2 × 2 × 2 supercell (see Experimental Section for more details). The parent Cu_3_Cl_4_(OH)_2_·2H_2_O structure contains four crystallographically distinct Cu sites. To assess the site preference and energetic impact of Zn substitution, we generated four Cu_2.5_Zn_0.5_Cl_4_(OH)_2_·2H_2_O structures in which Zn replaced each Cu site respectively (e.g., Zn1 = Zn@Cu1). Comparing the resulting formation energies from the relaxed DFT calculations reveals that among the substituted structures, Zn substitution on the Cu1 site yields the lowest formation energy, computed relative to the elemental DFT free energies from the Materials Project,^34,35^ consistent with the SCXRD results. To further investigate Zn stabilization in (Cu,Zn)_3_Cl_4_(OH)_2_·2H_2_O, we enumerated the 2 × 2 × 2 supercell with partial occupancies on the Cu1 site and performed spin-polarized DFT relaxations on the symmetrically unique structures. The convex hull (Figure 4b), which is the stability line connecting the most thermodynamically favored Cu_3–*x*_Zn_*x*_Cl_4_(OH)_2_·2H_2_O compositions, indicates a monotonic energetic preference for Zn substitution at the Cu1 site as the Zn fraction (*x*) increases. Within the limitations of the present DFT framework, compositions that lie on the convex hull are predicted to be thermodynamically stable at 0 K relative to the competing compositions, while those slightly above the hull are metastable. The lowest Zn fraction (*x*) for the Cu_3–*x*_Zn_*x*_Cl_4_(OH)_2_·2H_2_O structures lying on the convex hull is *x* = 0.3, approximately twice the Zn concentration determined by EDS (*x* = 0.15). Due to the absence of experimental information on the magnetic propagation vector and the simplified magnetic ordering, and the computational constraints inherent to DFT, the compositional resolution that can be modeled is limited and thus, we cannot further comment on the onset of stability. Overall, the DFT calculations corroborate the preferential occupation of Zn at the Cu1 site and reveal a consistent energetic trend with the increasing Zn content in Cu_3–*x*_Zn_*x*_Cl_4_(OH)_2_·2H_2_O.

**Figure 4:**
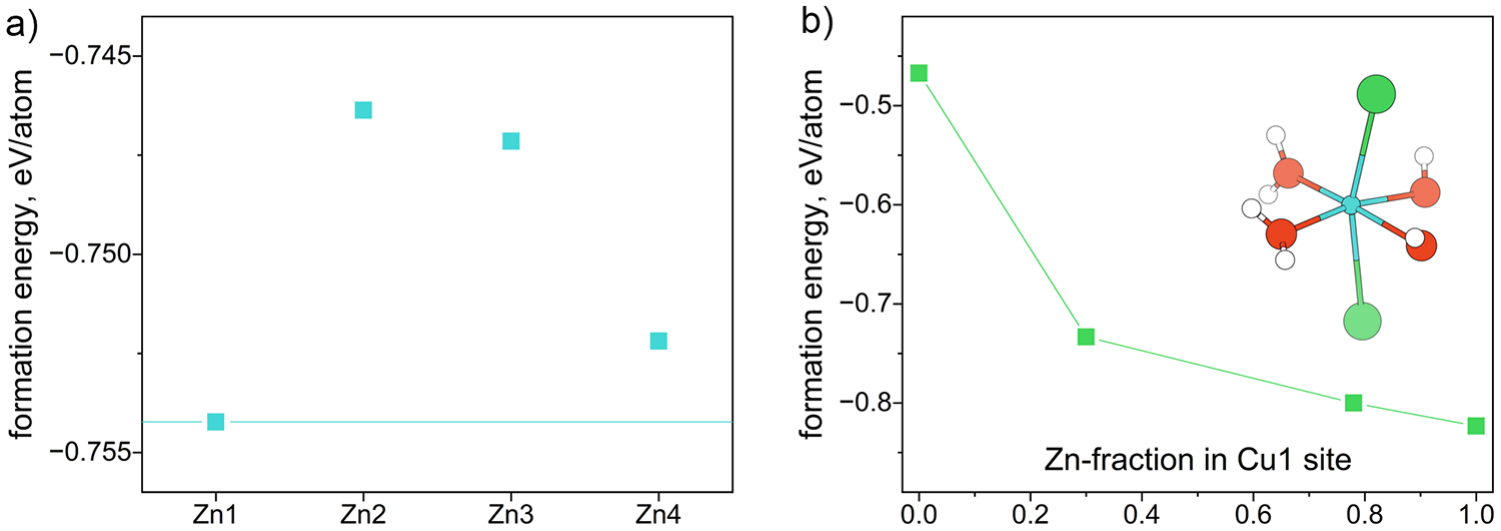
(a) Formation energies of the Cu_2.5_Zn_0.5_Cl_4_(OH)_2_·2H_2_O structures with Zn only on Cu1, Cu2, Cu3, and Cu4 sites. (b) Formation energies of Cu_3–*x*_Zn_*x*_Cl_4_(OH)_2_·2H_2_O structures, where *x* is Zn fraction or Zn occupancy on the Cu1 site. Note: only Cu_3–*x*_Zn_*x*_Cl_4_(OH)_2_·2H_2_O compositions that are on the convex hull are shown on the graph. The inset shows Cu1 site coordination environment. White, red, green, and blue spheres represent H, O, Cl, and Cu atoms, respectively.

Magnetosynthesis of (Cu,Zn)_3_Cl_4_(OH)_2_·2H_2_O crystals with a 0.19 T applied field (Scheme 1b) resulted in the same product with lattice parameters differing by approximately 0.2–1% (Table S6). This level of difference is consistent with literature reports of magnetosynthesis in iridates and ruthenates,^7,9^ although additional statistics may be needed to unambiguously confirm that application of a magnetic field during synthesis is the source of these differences instead of, perhaps, subtle differences in the crystals’ composition or temperature during measurement.

We speculated that magnetic field would most affect the magnetic Cu^2+^ ions. Comparison of Cu coordination environment between crystals synthesized with and without applied magnetic field revealed that the site with the largest differences is Cu1 (Figure 5a and Tables S7–S10). This is the same site that can be partially substituted by Zn, making all comparisons more ambiguous due to potential difference in Cu/Zn ration at the Cu1 site. The changes in the bond distances and angles in the Cu1 octahedron span within 0–0.5%, with bigger changes outside of 3σ range (Tables S9 and S10). This change in Cu1 coordination propagates to a Cu1 shift within the Cu network. However, as Cu1 is also the site that we hypothesize can accommodate Zn^2+^ most easily (as discussed above), these changes might also be due to slightly different Zn incorporation levels in different crystals.

**Figure 5:**
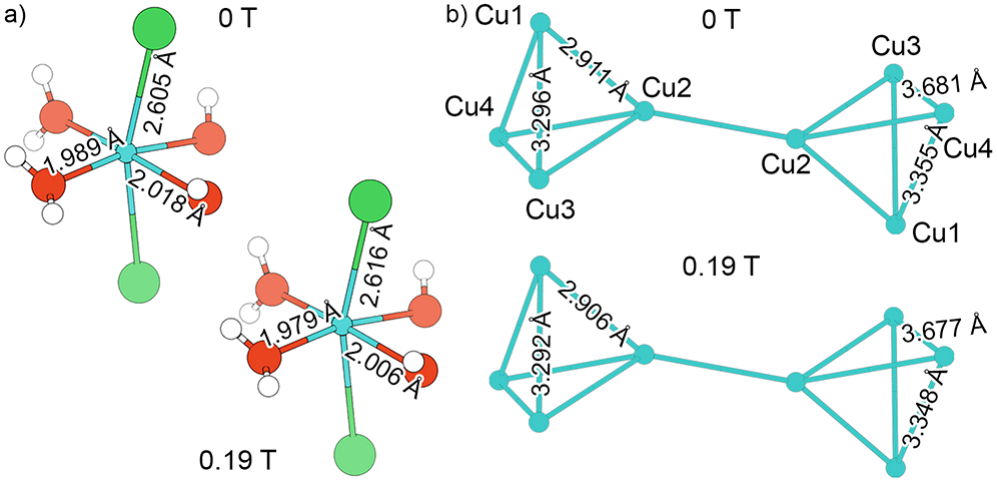
View of (a) Cu1 octahedra and (b) Cu network. Interatomic distances are shown for the most significant change (>0.10%) between the (Cu,Zn)_3_Cl_4_(OH)_2_·2H_2_O crystal structures solved at 130 K and grown with and without a 0.19 T field. White, red, green, and blue spheres represent H, O, Cl, and Cu atoms, respectively.

The Cu atoms themselves form an interesting lattice, shown below in Figure 3d,e. This lattice has stretched octagons in roughly the (011) plane formed by six tetrahedra (Figure 3d). These tetrahedra are highly distorted, as shown in Figure 5b. From the side, these distorted octagons stack as layers of interconnected bowties formed by Cu tetrahedra (Figure 5b). The Cu1–Cu2–Cu4 and Cu1–Cu3–Cu4 triangles are affected the most by magnetic field, and they shrink by 0.1–0.2%; this change is in the 2σ range (Table S9).

### Magnetic Properties of the (Cu,Zn)_3_Cl_4_(OH)_2_·2H_2_O Phase

We performed a Rietveld refinement on PXRD data of several ground (Cu,Zn)_3_Cl_4_(OH)_2_·2H_2_O crystals synthesized without an applied magnetic field (Figures 6a and S9; Tables S11 and S12). The structural model fit the data well (*R*_w_ = 3.40%), and no impurity phases were observed. We note that one prior report of polycrystalline Cu_3_Cl_4_(OH)_2_·2H_2_O contained CuCl_2_·2H_2_O impurities, which are not present in our sample.^13^

**Figure 6:**
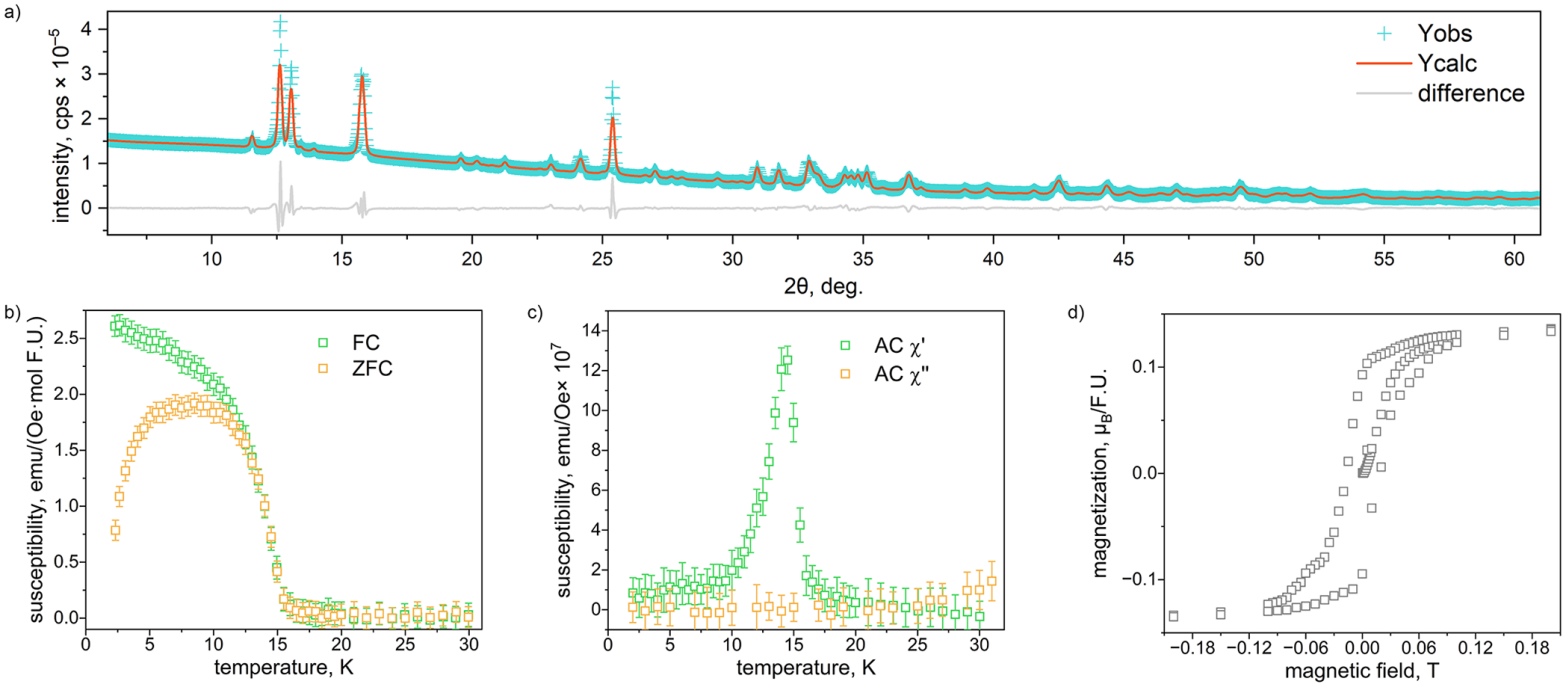
(a) Rietveld refinement of PXRD data of a few (Cu,Zn)_3_Cl_4_(OH)_2_·2H_2_O crystals. (b) ZFC and FC DC susceptibility measured at 0.005 T, (c) real and imaginary components of AC susceptibility measured at 0 T, and (d) field-dependent magnetization at 2 K for a few (Cu,Zn)_3_Cl_4_(OH)_2_·2H_2_O crystals. Note: the error bars in panel (d) are smaller than the square size.

We postulate that the magnetic behavior of (Cu,Zn)_3_Cl_4_(OH)_2_·2H_2_O is highly correlated to the type and level of structural distortion in the lattice. Therefore, we investigated the magnetic properties of this compound by measuring the magnetic susceptibility of a collection of small crystals of (Cu,Zn)_3_Cl_4_(OH)_2_·2H_2_O totaling ∼1 mg. We note that it was difficult to obtain a large enough quantity of these crystals for high quality measurements; in fact, we could not harvest even 1 mg of (Cu,Zn)_3_Cl_4_(OH)_2_·2H_2_O_0.19T crystallized under a magnetic field and therefore could not perform PXRD or magnetic characterization.

As Asaf et al. found for Cu_3_Cl_4_(OH)_2_·2H_2_O,^13^ the susceptibility data of (Cu,Zn)_3_Cl_4_(OH)_2_·2H_2_O display antiferromagnetic ordering at low temperature, with some hysteresis and a small net moment likely due to canting of the spins (Figure 6b). We observe a broad peak in the temperature-dependent susceptibility data collected at low field (μ_0_ *H* = 0.005 T, Figure 6a) with splitting between the ZFC and FC data, which may indicate some spin glass character. Based on both DC magnetization and AC susceptibility measurements (Figure 6b,c, respectively), the *T*_N_ of (Cu,Zn)_3_Cl_4_(OH)_2_·2H_2_O is approximately 15.5 K, slightly lower than the value of 17.5 K reported for Cu_3_Cl_4_(OH)_2_·2H_2_O;^13^ this is reasonable given the substitution of nonmagnetic Zn^2+^ for magnetic Cu^2+^. The field-dependent data show a small hysteresis loop with a small net moment that is likely due to canting of the spins (Figure 6d). Due to the tiny amount of sample, there was a large diamagnetic component in the data from the sample holder (Figure S21), and a linear Curie–Weiss fit was not possible even with a diamagnetic correction (χ_0_).

### Magnetosynthesis of CuCl_2_·2H_2_O

Since we were not able to investigate the effect of magnetosynthesis on the magnetic properties of the frustrated (Cu,Zn)_3_Cl_4_(OH)_2_·2H_2_O structure due to low synthetic yield, our next effort to probe the effect of magnetosynthesis on 3*d* ^9^ (*S* = 1/2) electronic systems focused on the room temperature evaporative crystallization of CuCl_2_·2H_2_O.

The Cu-only solution obtained via hydrothermal CuO/CuCl_2_ dissolution in HCl was allowed to evaporate under no magnetic field and fields of 0.19 and 0.37 T to obtain blue powders of CuCl_2_·2H_2_O (Scheme 1c, Figure 2). The phase purity of all CuCl_2_·2H_2_O samples was confirmed by PXRD. We performed a Rietveld refinement of the data collected at room temperature and found good agreement with the reported structure in *Pmna* space group (*R*_w_ = 2.22, 2.59, and 2.60% for 0, 0.19, and 0.37 T, respectively; Figures S13–S15; Tables S13 and S14); no additional phases were indexed. The changes in the unit cell for compositions synthesized under different applied fields vary and are larger than 2σ. The unit cell volume increases with increasing magnetic field, which may be related to the Jahn–Teller effect on the [CuCl_4_(H_2_O)_2_] octahedra.

The CuCl_2_·2H_2_O structure consists of edge-sharing [CuCl_4_(H_2_O)_2_] octahedral chains with terminal water molecules (Figure 7a,b). The Cu network consists of linear chains (Figure 7c) aligned along (0 0 1), indicating no prerequisite for frustration based on triangular geometry. The DC susceptibility data of all three samples (0, 0.19, and 0.37 T) revealed a broad AFM peak at around 5.8 K (Figures S22, S23, and Table S18) with no significant discrepancy between ZFC and FC data (Figure S22). This matches the literature which lists the AFM transition at 4.3 K (inflection point).^10^ The real part of the AC susceptibility (χ’, Figure S22) for all three samples demonstrated a broad peak centered at 5.7 K, matching the DC magnetization data.

**Figure 7:**
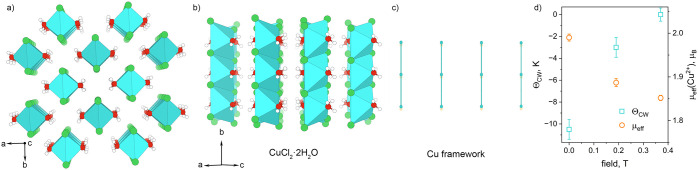
View of the CuCl_2_·2H_2_O structure along (a) [001] and (b) [101] and (c) Cu framework. White, red, green, and blue spheres and blue polyhedra represent H, O, Cl, and Cu atoms, and Cu octahedra, respectively. (d) Parameters extracted from Curie–Weiss fits of CuCl_2_·2H_2_O synthesized under varying magnetic fields.

As shown in Figure S22, there is no apparent difference in the magnetic susceptibility of CuCl_2_·2H_2_O synthesized under varying magnetic field strengths. Each curve was smoothed with a weighted adjacent-averaging algorithm, and the peak maximum was determined as the first derivative plot crossing *y* = 0. These results are shown in Table S18 and indicate that there is no significant difference in AFM peak between samples. Due to the broad nature of the AFM transition in CuCl_2_·2H_2_O, it is hard to quantitatively compare Néel temperature or inflection point between the three samples.

The high temperature magnetic susceptibility data were fit to a Curie–Weiss model from 100–310 K (Figure S24). As shown in Figure 7d, synthesis under a magnetic field influenced Θ_CW_; the values increase (i.e., become less AFM) with the field applied during the synthesis (Table S18). A small decrease in the effective moment μ_eff_ per Cu^2+^ is observed with increasing magnetic field as well. To probe the significance of these changes, we performed a stability analysis of the Curie–Weiss fits across temperature ranges and with various diamagnetic corrections (χ_0_, Table S19), finding that these fits are sensitive to χ_0_. Fits in the 150–310 K temperature range demonstrated similar trends, but fits from 200–310 K were unstable when χ_0_ was refined freely, likely due to the smaller number of data points; fixing χ_0_ to the value from the 100–310 K fit yielded the same trends of increasing Θ_CW_ and decreasing μ_eff_. These results suggest that magnetosynthesis may be suppressing the AFM interactions as well as the magnetic moment of Cu^2+^ in CuCl_2_·2H_2_O.

### Magnetosynthesis of Atacamite Cu_2_(OH)_3_Cl

Finally, our attempts to synthesize Cu_3_Cl_4_(OH)_2_·2H_2_O via the published CuCl_2_·2H_2_O rehydration methods yielded instead a mixture of Cu_3_Cl_4_(OH)_2_·2H_2_O and atacamite Cu_2_(OH)_3_Cl.^13^ Over the course of several weeks, Cu_3_Cl_4_(OH)_2_·2H_2_O transformed to atacamite Cu_2_(OH)_3_Cl (Figure S2), confirming the metastability of Cu_3_Cl_4_(OH)_2_·2H_2_O. Note that initial synthesis of atacamite was performed in summer; our attempts to reproduce the synthesis in winter resulted mostly in CuCl_2_·2H_2_O. This suggests that phase transformations between metastable Cu_3_Cl_4_(OH)_2_·2H_2_O, Cu_2_(OH)_3_Cl, CuCl_2_·2H_2_O, and Cu­(OH)Cl are temperature- and humidity-dependent. Therefore, future efforts to develop a robust recipe to crystallize one of these compounds must carefully optimize and control temperature and humidity.

Atacamite Cu_2_(OH)_3_Cl has an orthorhombic structure with a distorted triangular lattice of Cu^2+^ forming a weakly coupled 3D network of anisotropic sawtooth chains,^14^ which leads to geometric magnetic frustration (Figure 8a,b). The low temperature magnetic behavior of both natural and synthetic atacamite have been the subject of prolonged interest and debate: it exhibits a magnetic transition to an AFM but likely disordered and/or spin-glassy ground state.^15,16,18,19,42,43^ While different ordering temperatures of *T*_N_ ≈ 5.8 K for synthetic atacamite^15,16^ and *T*_N_ ≈ 9 K for natural atacamite^18,19^ have been reported, both materials are highly frustrated *S* = 1/2 quantum magnets (*f* ≈ 17 for synthetic samples^16^). Differences in magnetic properties between natural and synthetic minerals are not surprising or uncommon, as natural minerals often have different and/or much higher levels of impurities than synthetic samples.

**Figure 8:**
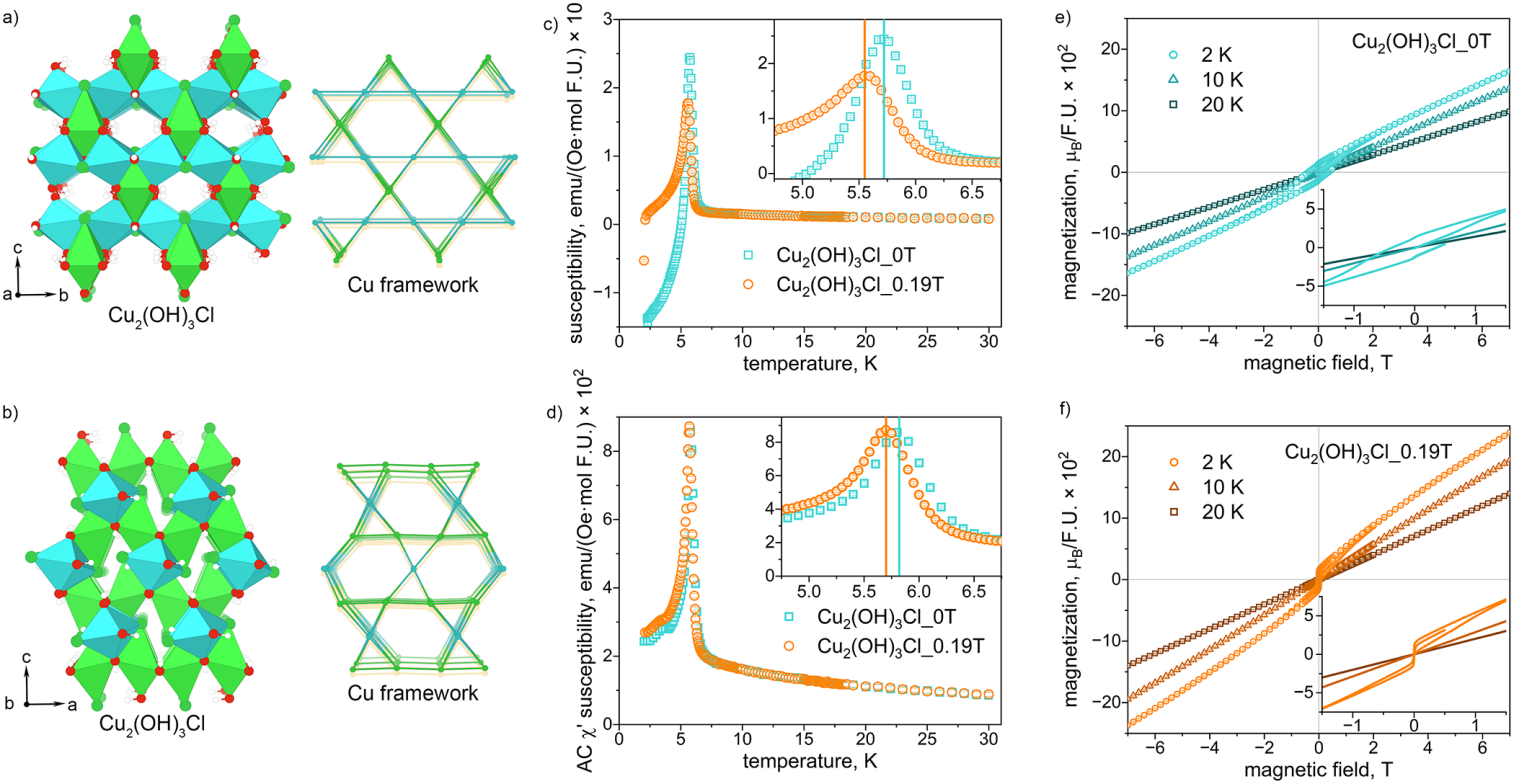
View of the Cu_2_(OH)_3_Cl atacamite structure and Cu framework along (a) [001] and (b) [010] directions. White, red, dark green, and blue and green polyhedra represent H, O, and Cl atoms, and two sites for Cu octahedra, respectively. (c) ZFC DC susceptibility measured at 0.005 T and (d) real component of the AC susceptibility measured at 0 T for Cu_2_(OH)_3_Cl synthesized under various magnetic field strengths: 0 and 0.19 T, respectively. Magnetization as a function of applied field at 2 K, 10 and 20 K for (e) Cu_2_(OH)_3_Cl_0T and (f) Cu_2_(OH)_3_Cl_0.19T samples.

We synthesized polycrystalline atacamite in both no magnetic field and an applied field of 0.19 T (Scheme 1d). While these samples were not highly crystalline due to the synthetic process, we performed Rietveld fits of PXRD data collected at room temperature (Figures S16 and S17). The samples synthesized with both 0 and 0.19 T fields yielded pure atacamite Cu_2_(OH)_3_Cl (*R*_w_ = 4.12% and *R*_w_ = 4.63%, respectively, Tables S15 and S16). The lattice parameter and bond distance differences between the Cu_2_(OH)_3_Cl_0T and Cu_2_(OH)_3_Cl_0.19T samples are mostly within the 1σ range (Table S15) except the *a* parameter; however, due to noticeably larger peak widths for atacamite compared to the other materials studied here, we treat this change in *a* more skeptically. EDS analysis on the atacamite powders resulted in similar Cu to Cl ratio of 2:1.19 and 2:1.17 for Cu_2_(OH)_3_Cl_0T and Cu_2_(OH)_3_Cl_0.19T, respectively (Figures S6 and S7).

AC susceptibility and DC magnetization measurements (Figures 8c,d and S27) on these two samples show a sharp peak at approximately *T*_N_ ≈ 5.7 K, consistent with previous data on synthetic atacamite.^15,16^ Intriguingly, the transition shifts by approximately 0.15 K between the 0 and 0.19 T samples (Figures 8c,d and S27; Table S20). This is evident in both DC and AC χ’ data, indicating that magnetosynthesis might have affected the magnetic ground state. While subtle, this 0.15 K difference is statistically significant: the measurement step size in the region of *T*_N_ was 0.05 K, with an associated error of ∼0.025 K. Frequency-dependent AC susceptibility measurements performed with zero applied field on both Cu_2_(OH)_3_Cl_0T and Cu_2_(OH)_3_Cl_0.19T samples (Figure S28) display a shift in the *T*_N_ to higher temperature with increasing frequency, confirming spin glass character of the magnetic ground state that was reported previously for synthetic atacamite.^15,16^

To prove that the 0.15 K difference in *T*_N_ is not a measurement artifact, we performed the DC magnetization measurement on the Cu_2_(OH)_3_Cl_0T sample twice and derived the same *T*_N_ = 5.72 K from both data sets (Figure S25). Moreover, we compared two aliquots of Cu_2_(OH)_3_Cl_0.19T taken from the same batch and washed with acetone 2 months apart (Figure S26). The first aliquot contained a Cu_3_Cl_4_(OH)_2_·2H_2_O impurity identified at 0.3 wt.% level supported by PXRD and EDS (Figure S26). The second aliquot did not contain a Cu_3_Cl_4_(OH)_2_·2H_2_O impurity, which likely decomposed over time; at the same time, the Cu_2_(OH)_3_Cl particles were significantly smaller due to recrystallization of Cu_2_(OH)_3_Cl (Figure S26). Nonetheless, both Cu_2_(OH)_3_Cl_0.19T data sets yield the same 5.55 K *T*_N_ (Figure S26). Those experiments demonstrated that *T*_N_ for Cu_2_(OH)_3_Cl samples is independent of the PPMS sample loading, Cu_2_(OH)_3_Cl particle size, or the presence of a Cu_3_Cl_4_(OH)_2_·2H_2_O impurity.

We performed Curie–Weiss fits from 150–350 K on DC magnetization data collected under an applied field of μ_0_ *H* = 1 T (see Figure S30, Tables S20, and S21) and found Weiss temperatures Θ_CW_ of −73(5) and −68(3) K for the 0 and 0.19 T samples, respectively, confirming the overall AFM nature of the magnetic interactions and the magnetic frustration of these samples (frustration index *f* ≈ 12). We note that while the extracted μ_eff_ per Cu^2+^ are lower than the expected value, they depend strongly on fit range and diamagnetic correction; they are consistent between samples within error.

The magnetization as a function of applied field at *T* = 2 K (Figure 8e,f) exhibit clear yet small hysteresis loops, although Cu_2_(OH)_3_Cl_0T is noticeably harder than Cu_2_(OH)_3_Cl_0.19T; the samples have small net moments of 0.013 and 0.006 μ_B_/mol f.u. for the 0 and 0.19 T samples, respectively. The coercive fields are approximately 0.38 and 0.003 T, respectively. Both samples display a small amount of wasp-waisted behavior, suggesting the coexistence of at least two competing interactions. We observe that the magnetization at high field is consistently higher in Cu_2_(OH)_3_Cl_0.19T at all temperatures measured (Figure 8e,f), which may be consistent with either a different intrinsic magnetic structure or better alignment of particles under an applied field. Neutron scattering data would be key to resolving this open question. Taken together, the isothermal magnetization behavior is consistent with canted AFM ground states in both samples, albeit with slight differences below onset of magnetic order, consistent with the temperature-dependent data.

## Discussion

As discussed above, this is one of the first studies on magnetosynthesis in 3*d* systems, and the first on a 3*d* insulating system (recent work has been performed on synthesizing metallic Co out of a Co–S flux).^44,45^ The effects we see here seem much weaker than those observed in the (still few) 4*d* and 5*d* systems that have been studied so far.^7–9^ This may be consistent with the hypothesis of Cao et al. that strong spin–orbit interactions can yield large structural and magnetic changes with magnetosynthesis,^7^ although we note that the ∼0.2–0.5% lattice changes we observe here are smaller but on the same order of magnitude reported for, e.g., BaIrO_3_ (∼0.7–0.85%).^9^ This is therefore worthy of further theoretical study.

Overall, the only material out of the four studied here that showed a shift in magnetic properties with magnetosynthesis is atacamite, which exhibits strong frustration and a slightly canted AFM ground state. The ∼3% shift in Néel temperature between the 0 and 0.19 T samples is subtle yet robust. (Cu,Zn)_3_Cl_4_(OH)_2_·2H_2_O, which has a canted AFM ground state with a small net moment, exhibited changes in lattice parameters and coordination of Cu^2+^ cations. These changes might correlate with the presence of a magnetic field during crystal growth or with local discrepancies in composition (i.e., the Cu:Zn ratio on Cu1 site); ultimately, such a complex, low-symmetry (*P*-1) structure is likely not an ideal candidate for this type of study. Unfortunately, we were not able to synthesize enough material under an applied field to compare magnetic properties. Moreover, the occupancy of Zn at the Cu1 site as a function of field can affect magnetic properties yet is challenging to determine without resonant single crystal X-ray diffraction, which requires a synchrotron, or growing crystals large enough for neutron diffraction.

Interestingly, the simple AFM (CuCl_2_·2H_2_O) and the QSL (HBS) did not exhibit a significant difference in magnetic properties (*T*_N_ for CuCl_2_·2H_2_O and Θ_CW_ for both materials). While this was complicated by the broadness or lack of peaks in the susceptibility data and by the known variability in Curie–Weiss fits, we speculate that CuCl_2_·2H_2_O’s AFM ground state is likely too stable for the energy scale of a small magnetic field (∼0.02 meV per *S* = 1/2 for the 0.19 T field to ∼0.037 meV for the 0.37 T field) to perturb. We postulate that the degenerate frustrated AFM ground states—that also exhibit tiny net moments, likely due to spin canting—of (Cu,Zn)_3_Cl_4_(OH)_2_·2H_2_O and atacamite may be more accessible to these small energy scales. However, HBS, which is much more frustrated than the other materials, was not apparently affected, albeit at a lower synthesis field of 0.09 T. HBS’s AFM interactions are the strongest (*J* ≈ 15 meV) out of all materials studied here, and its spin gap (∼1 meV) far exceeds the ∼0.01 meV per *S* = 1/2 scale of our magnetosynthesis. This tentatively points to a middle range of magnetic frustration more affected by magnetosynthesis than a nonfrustrated sample or a fully frustrated QSL.

There are many open questions about the possible interplay of magnetic fields with synthetic factors such as phase purity, speciation, composition, morphology, etc., as well as how all these factors influence the resulting structure and physical properties. Once these factors are rigorously controlled, we hypothesize that magnetosynthesis may affect the magnetic ground state in several ways: (1) it may induce a magnetostructural effect that influences the exchange interactions, which is borne out by the structural changes observed in (Cu,Zn)_3_Cl_4_(OH)_2_·2H_2_O, and (2) it may “select” or stabilize a magnetic configuration with either competing interactions and/or a net moment (like we showed here with atacamite). Further study, likely involving developing novel computational techniques, will be needed to fully investigate these hypotheses and how universal they may be.

While discussing the results presented above, we attempted to maintain a skeptical attitude toward subtle changes in the structure and magnetic properties in relation to the introduction of a magnetic field in the synthesis. These studies turned out to be very challenging, especially considering the metastable nature of these phases, which influenced reproducibility and affected the crystal structure and hence properties. We believe that future studies should focus on 3*d*-containing thermodynamically stable phases as well as metastable 4*d*- and 5*d*-based compounds, to fully understand how thermodynamic and kinetic stabilities interact with spin-only and spin–orbit coupling in magnetosynthesis.

The low-temperature methods that we develop here for incorporating a magnetic field during synthesis are novel; previous work placed permanent magnets outside a box furnace, severely limiting the strength that can be achieved. Thus, these methods can easily be applied to other materials families to continue exploring the use of magnetic field as a synthetic handle. In addition, detailed studies of transport properties, electron paramagnetic resonance spectroscopy, Raman vibrational modes, and photophysics would also help to unravel the effects of magnetosynthesis on many variables, including local structure, speciation of complexes, and ion anisotropy, etc.

## Conclusions

Magnetic field is a highly underexplored synthetic variable, and most work studying its effect has been performed on 4*d* and 5*d* transition metal-containing compounds. Here, we performed the first systematic exploration of magnetosynthesis in 3*d* compounds, focusing on materials containing *S* = 1/2 Cu^2+^ in a variety of lattices and with a range of magnetic frustration. We developed novel methods to easily incorporate a magnetic field into low-temperature hydrothermal and room-temperature evaporative and rehydration synthesis techniques. We applied these methods to a series of materials that exhibit a range of low-temperature magnetic properties from QSL (HBS Cu_3_Zn­(OH)_6_Cl_2_) to simple low-temperature antiferromagnetism (CuCl_2_·2H_2_O) to complex low-temperature antiferromagnetism with spin canting and/or spin-glass behavior ((Cu,Zn)_3_Cl_4_(OH)_2_·2H_2_O and atacamite Cu_2_(OH)_3_Cl). Intriguingly, atacamite Cu_2_(OH)_3_Cl exhibited a 0.15 K (∼3%) decrease of its *T*_N_ with magnetosynthesis under a 0.19 T field, suggesting that magnetosynthesis may affect the magnetic properties of frustrated quantum materials with 3*d* transition metals.

Notably, we observed the stabilization of an understudied phase Cu_3_Cl_4_(OH)_2_·2H_2_O by incorporating a small amount of Zn (Cu:Zn 2.85:0.15), confirmed this with DFT calculations, and report the first single crystal structural determination of this structure type as well as the magnetic properties of this phase. Its Cu/Zn lattice consists of a stretched and highly distorted kagome arrangement, with three out of the four Cu/Zn sites in distorted octahedral geometry and the last in distorted square pyramidal geometry. We investigated whether synthesis of this phase under a 0.19 T magnetic field well above the magnetic ordering temperature of ∼15 K would result in structural changes; however, we could not deconvolute the effects of field from possible Cu/Zn differences. Cu_3.85_Zn_0.15_Cl_4_(OH)_2_·2H_2_O exhibits a magnetic transition at *T*_N_ ≈ 15.5 K with a small net moment below this transition, suggesting a canted antiferromagnetic ground state.

## Supplementary Material



## Notes

Synthetic details; scanning electron microscopy data; crystallographic details of the SCXRD structures; PXRD data and crystallographic data; and additional magnetism data (PDF)

## §

Materials Science & Engineering, University of Colorado Boulder, Boulder, Colorado 80309, United States
