# Supplementary material for: Magnetosynthesis Effect on the Structure and Ground State of Cu2+-Based Antiferromagnets
Source: Inorg Chem. 2026 Apr 1;65(14):7636–48. doi: 10.1021/acs.inorgchem.5c05555 (PMC13080985; doi:10.1021/acs.inorgchem.5c05555)
Supplement: Supplementary file 1 [file ic5c05555_si_001.pdf]

Supporting Information:

Magnetosynthesis effect on the structure and ground state of Cu^{2+}-based antiferromagnets

Micaela E. Primer,[a,#,†] Anna A. Berseneva,[a,#,] Ayesha Ulde,[b] Wenhao Sun,[b] Rebecca W. Smaha[a,*]*

[a] Materials Science Center, National Laboratory of the Rockies, Golden, Colorado, 80401, USA

[b] Department of Materials Science and Engineering, University of Michigan, Ann Arbor, Michigan, 48109, USA

[#] These authors contributed equally to this work.

* Anna.Berseneva@NLR.gov; Rebecca.Smaha@NLR.gov

Table of Contents

1. SYNTHETIC DETAILS 3
2. SCANNING ELECTRON MICROSCOPY 5
3. COORDINATION ENVIRONMENT ANALYSIS 7
4. CRYSTALLOGRAPHY AND DIFFRACTION 8
   a. Herbertsmithite $Cu_3Zn(OH)_6Cl_2$ 8
   b. $(Cu,Zn)_3Cl_4(OH)_2{\cdot}2H_2O$ 9
   c. $CuCl_2{\cdot}2H_2O$ 17
   d. Atacamite $Cu_2(OH)_3Cl$ 21
5. ADDITIONAL MAGNETIC DATA 24
REFERENCES 31

1. SYNTHETIC DETAILS

Table S1. The HBS reaction details: reagents, yield, and composition.

attempt	Cu:Zn ratio	$Cu_2(OH)_2CO_3$, mmol & g	CuO, mmol & g	$ZnCl_2$, mmol & g	H_2O, mL	yield, %	CuO impurity
**1**	2.6:1	2.98 & 0.66	--	2.27 & 0.31	10	88%	Yes
**2**	2.0:1	2.26 & 0.50	--	2.27 & 0.31	10	83%	Yes
**3**	1.2:1	1.36 & 0.40	--	2.28 & 0.31	10	87%	Yes
**4**	0.6:1	1.35 & 0.30	--	4.42 & 0.60	10	79%	No
**5**	0.3:1	0.90 & 0.20	--	5.52 & 0.75	10	34%	No
**1'**	0.16:1	--	1.00 & 0.08	5.94 & 0.81	10	81%	Yes
**2'**	0.08:1	--	0.50 & 0.04	6.10 & 0.83	10	82%	Yes
**3'**	0.04:1	--	0.26 & 0.02	6.60 & 0.90	10	5%	No
**4'**	0.02:1	--	0.13 & 0.01	6.58 & 0.90	10	34%	No

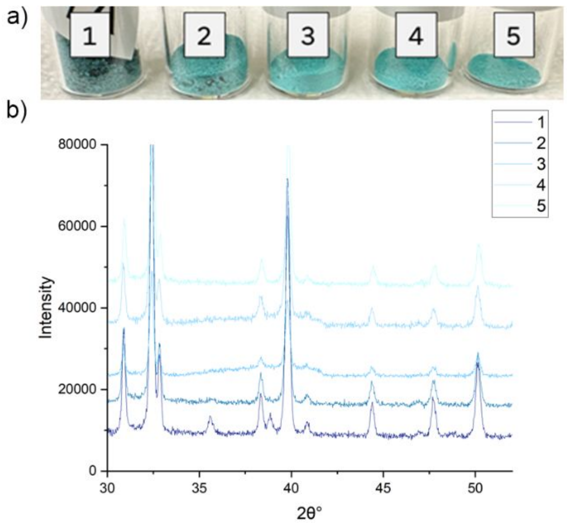

Figure S1. (*a*) Visual inspection and (*b*) PXRD patterns for HBS synthesis attempts 1-5. The data show the CuO impurity in samples 1 and 2 (at ~35.5 and ~38.8 deg.).

Table S2. Evaporative crystallization reaction attempts with Cu, Zn, and both Cu and Zn.

**Cu**	CuO<br>0.50 mmol<br>40 mg	$CuCl_2 \cdot 2H_2O$<br>4.87 mmol<br>830 mg	H_2O<br>9 ml	HCl<br>1 ml
**Zn**	ZnO<br>0.49 mmol<br>40 mg	$ZnCl_2$<br>6.09 mmol<br>830 mg	H_2O<br>9 ml	HCl<br>1 ml
**Cu/Zn**	CuO<br>0.50 mmol<br>40 mg	$ZnCl_2$<br>6.09 mmol<br>830 mg	H_2O<br>9 ml	HCl<br>1 ml

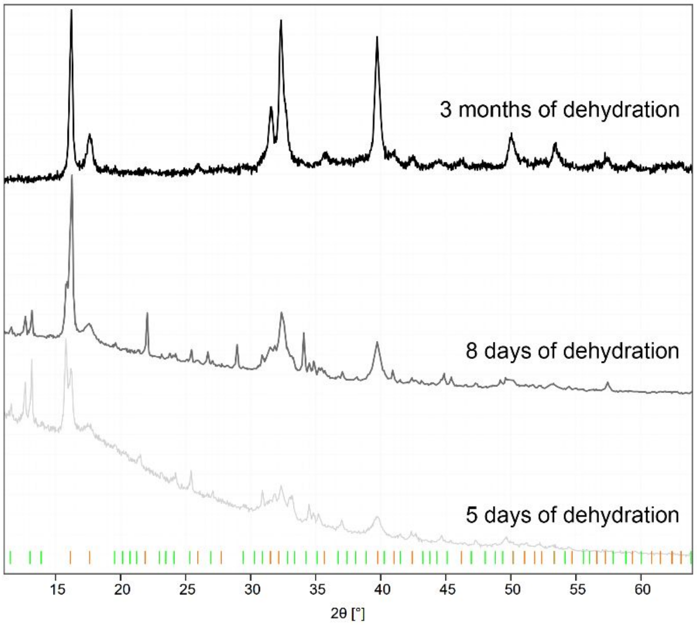

Figure S2. Synthesis of $Cu_3Cl_4(OH)_2 \cdot 2H_2O$ through dehydration/hydration cycle shown through laboratory PXRD data. Green and Orange ticks correspond to $Cu_3Cl_4(OH)_2 \cdot 2H_2O$ and $Cu_2(OH)_3Cl$, respectively.

2. SCANNING ELECTRON MICROSCOPY

SEM images of the blue and green crystals show that the blue crystal is more granular than the green (Figure S1). This supports the hypothesis of $(Cu,Zn)Cl_2 \cdot 2H_2O$ instability.

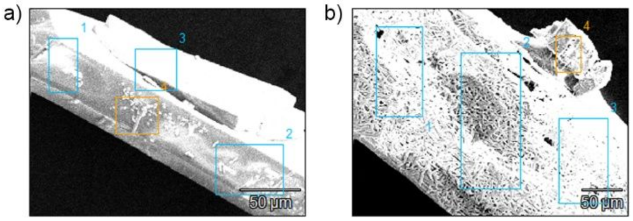

Figure S3. SEM images of (a) a green crystal of $(Cu,Zn)_3Cl_4(OH)_2 \cdot 2H_2O$ and (b) a blue crystal of $(Cu,Zn)Cl_2 \cdot 2H_2O$. According to the EDS spectra the Cu:Zn:Cl ratios are estimated at 2.59:0.41:28.14 and 0.86:0.14:14.66 for the (a) $(Cu,Zn)_3Cl_4(OH)_2 \cdot 2H_2O$ and (b) $(Cu,Zn)Cl_2 \cdot 2H_2O$ samples, respectively. The Zn and Cl content is likely exaggerated due to insufficient washing leaving $ZnCl_2$ salts on top of the crystal.

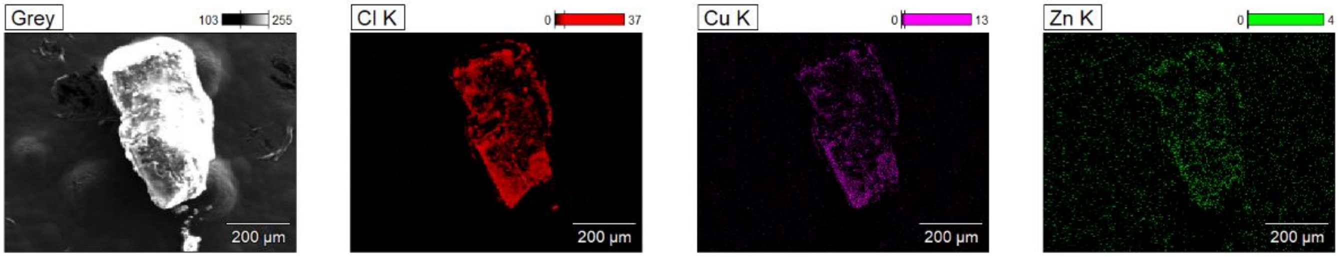

Figure S4. EDS mapping on the washed $(Cu,Zn)_3Cl_4(OH)_2 \cdot 2H_2O$_0T crystal.

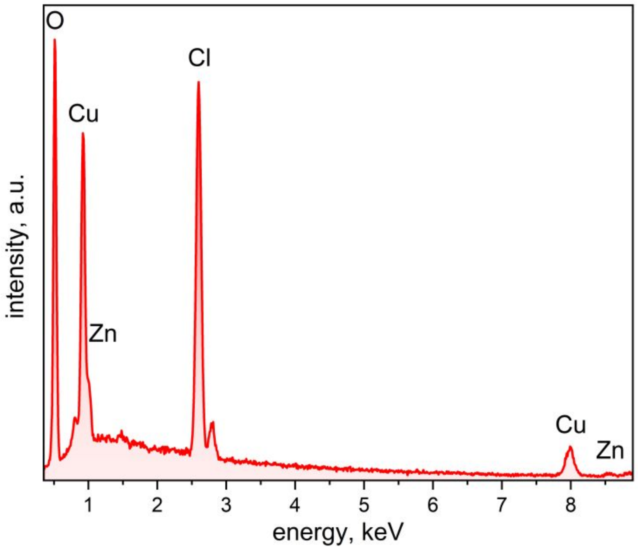

Figure S5. EDS spectrum of the washed $(Cu,Zn)_3Cl_4(OH)_2 \cdot 2H_2O$_0T crystal. The Cu:Zn:Cl ratio is estimated at 2.85:0.15:5.01.

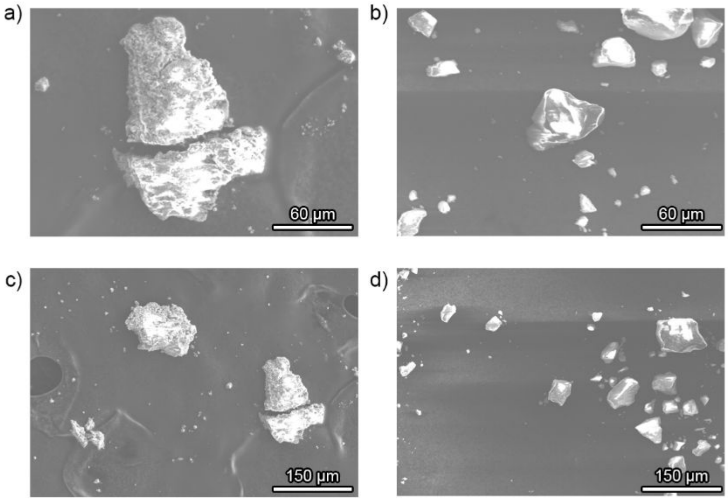

Figure S6. SEM images of $Cu_2(OH)_3Cl$_0T and $Cu_2(OH)_3Cl$_0.19T powder.

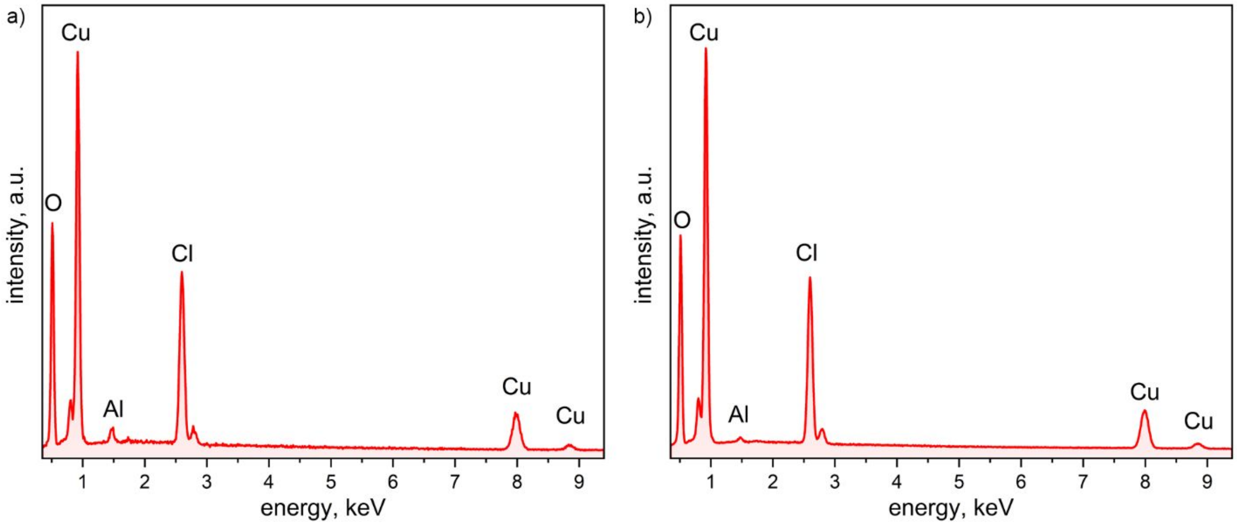

Figure S7. EDS spectra of $Cu_2(OH)_3Cl$_0T and $Cu_2(OH)_3Cl$_0.19T powders. The Cu:Cl ratios are estimated at 2:1.19 and 2:1.17, respectively. Note: Al impurities are always present due to the sample holder material.

3. COORDINATION ENVIRONMENT ANALYSIS

Table S3. Coordination environments of M^{2+} in unique M-Cl-O-H (M = Cu, Zn) compounds reported in the ICSD,[1] with their frequency in parentheses. Note that some compounds did not assign H, so MO_x may be equivalent to $M(OH)_x$ or $M(H_2O)_x$.

	**Octahedral (including distorted)**	**Trigonal prismatic**	**Square pyramidal**	**Tetrahedral**	**Square Planar**
**Cu^{2+}**	CuO_6 *(2)*<br>$Cu(H_2O)_6$ *(2)*<br>$Cu(OH)_6$ *(5)*<br>$CuO_2(H_2O)_4$ *(1)*<br>$Cu(OH)_5Cl$ *(2)*<br>CuO_4Cl_2 *(2)*<br>$Cu(OH)_4Cl_2$ *(7)*<br>$Cu(OH)_3Cl_3$ *(1)*<br>$Cu(H_2O)_2Cl_4$ *(1)*	$Cu(OH)_6$ *(1)*	$Cu(H_2O)_5$ *(1)*<br>$Cu(H_2O)_4Cl$ *(1)*	*Not observed*	CuO_4 *(1)*
**Zn^{2+}**	ZnO_6 *(2)*<br>$Zn(H_2O)_6$ *(2)*<br>$ZnO_4(H_2O)_2$ *(1)*<br>$ZnO_2(OH)_2(H_2O)_2$ *(1)*<br>$Zn(H_2O)_5Cl$ *(1)*<br>ZnO_4Cl_2 *(1)*<br>ZnO_3Cl_3 *(1)*	*Not observed*	*Not observed*	$ZnCl_4$ *(5)*<br>$ZnCl_3O$ *(1)*	*Not observed*

4. CRYSTALLOGRAPHY AND DIFFRACTION

a. Herbertsmithite $Cu_3Zn(OH)_6Cl_2$

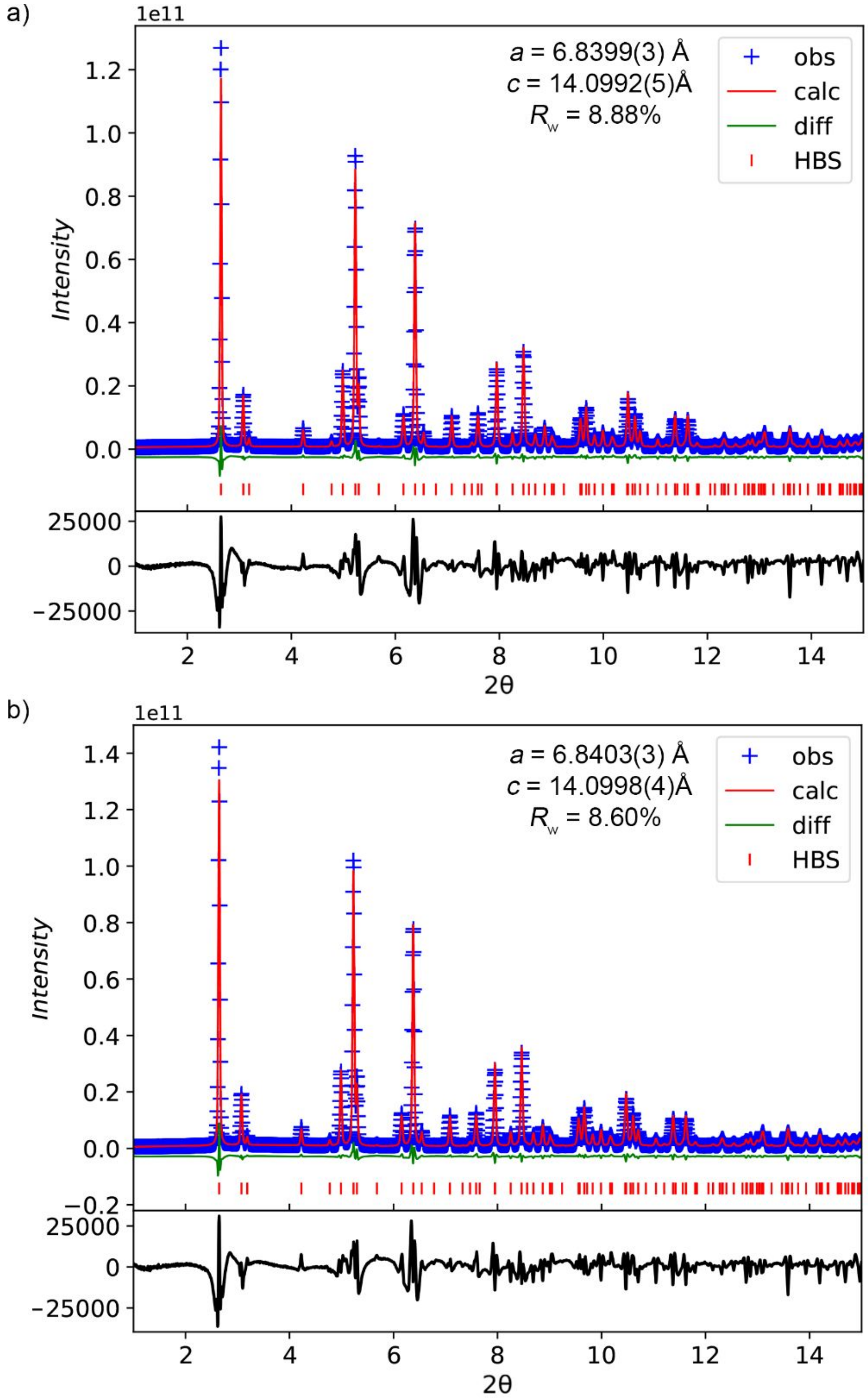

Figure S8. Rietveld refinements of synchrotron PXRD data for (*a*) HBS_0T and (*b*) HBS_0.09T.

Table S4. Parameters extracted from Rietveld refinements of synchrotron PXRD data of HBS in space group *R*-3*m* measured at room temperature. The difference between the structures grown with different applied fields is shown as $\Delta_{0.09-0\,T}$, with differences greater than the sum of the error bars in **bold**.

	**HBS_0T**	**HBS_0.09T**	$\Delta_{0.09-0\,T}$
***a* (Å)**	6.8399(3)	6.8403(3)	0.0004
***c* (Å)**	14.0992(5)	14.0999(4)	0.0007
**V (Å^3)**	571.25(5)	571.35(3)	**0.1**
**R (%)**	6.49	6.33	
**wR (%)**	8.88	8.60	
**Chi sq.**	5.49 x 10^{10}	5.88 x 10^{10}	
**Goodness of fit**	4701.9	4861.0	
**Preferred Orientation**	(0 0 1)	(0 0 1)	

Table S5: Fractional atomic coordinates and equivalent isotropic displacement parameters extracted from Rietveld refinements of synchrotron PXRD data of HBS in space group *R*-3*m* measured at room temperature.

	**Site**	**Wyckoff Position**	**x**	**y**	**z**	**occ**	B_{eq}
**0 T**	Cu1	-3m	0	0	0	0.53(7)	0.0093(2)
	Zn1	-3m	0	0	0	0.47(7)	0.0093(2)
	Cu2	2/m	0.83333	0.16667	0.16667	1	0.0093(2)
	Cl1	3m	0.66667	0.33333	0.0286(2)	1	0.0135(7)
	O1	m	0.130(1)	0.260(2)	0.1041(2)	1	0.0135(7)
**0.09 T**	Cu1	-3m	0	0	0	0.47(7)	0.0102(2)
	Zn1	-3m	0	0	0	0.53(7)	0.0102(2)
	Cu2	2/m	0.83333	0.16667	0.16667	1	0.0102(2)
	Cl1	3m	0.66667	0.33333	0.0286(2)	1	0.0153(7)
	O1	m	0.130(1)	0.260(2)	0.1042(2)	1	0.0153(7)

b. $(Cu,Zn)_3Cl_4(OH)_2 \cdot 2H_2O$

Table S6. Crystal data and structure refinement for $Cu_{2.85}Zn_{0.15}Cl_4(OH)_2(H_2O)_2$ collected with GaKα (λ = 1.34139) radiation at several temperatures and applied magnetic fields. The difference between the structures grown with different applied fields and collected at 130 K is shown as $\Delta_{\mathbf{0.19-0\,T}}$, with differences greater than the sum of the error bars in **bold**.

Temperature (K)	300	130	130	130
Applied field (T)	0	0	0.19	$\Delta_{\mathbf{0.19-0\,T}}$
Space group	*P*-1	*P*-1	*P*-1	
a (Å)	7.3837(15)	7.3613(3)	7.3539(3)	**-0.0074**
b (Å)	8.4458(18)	8.4497(3)	8.4478(3)	**-0.0019**
c (Å)	8.7357(18)	8.7278(3)	8.7355(3)	**0.0077**
α (°)	116.000(9)	116.3590(10)	116.3910(10)	**0.032**
β (°)	90.189(10)	90.180(2)	90.1780(10)	-0.002
γ (°)	113.611(9)	113.6160(10)	113.6540(10)	**0.038**
Volume ($Å^3$)	437.93(16)	434.73(3)	434.26(3)	**-0.47**
Z	2	2	2	
ρ_{calc} (g/cm^3)	3.054	3.077	3.080	
μ (mm^{-1})	44.773	44.604	44.652	
F(000)	386.0	386.0	386.0	
Crystal size (mm^3)	0.02 × 0.01 × 0.005	0.1 × 0.015 × 0.015	Not recorded	
2θ range	10.04 to 146.802	10.088 to 127.2	10.084 to 126.904	
Index ranges	$-10 \leq h \leq 8$, $-11 \leq k \leq 12$, $-12 \leq l \leq 12$	$-9 \leq h \leq 8$, $-11 \leq k \leq 11$, $-11 \leq l \leq 11$	$-9 \leq h \leq 9$, $-11 \leq k \leq 11$, $-11 \leq l \leq 11$	
Refl. collected	11890	6316	16527	
Indep. reflections	2607 [R_{int} = 0.0437, R_{sigma} = 0.0360]	2143 [R_{int} = 0.0598, R_{sigma} = 0.0636]	2120 [R_{int} = 0.0427, R_{sigma} = 0.0262]	
Data/restraints/ parameters	2607/4/117	2143/0/104	2120/0/106	
Goodness-of-fit on F^2	1.083	1.061	1.134	
Final R indexes [I>=2σ (I)]	R_1 = 0.0316 wR_2 = 0.0699	R_1 = 0.0615 wR_2 = 0.1653	R_1 = 0.0401 wR_2 = 0.1146	
Final R indexes [all data]	R_1 = 0.0429 wR_2 = 0.0728	R_1 = 0.0638 wR_2 = 0.1682	R_1 = 0.0409 wR_2 = 0.1168	
Largest diff. peak/hole (e $Å^{-3}$)	0.58/-0.74	1.97/-1.85	1.41/-1.28	

Table S7. Fractional atomic coordinates and equivalent isotropic displacement parameters ($Å^2×10^3$) from SCXRD. U_{eq} is defined as 1/3 of the trace of the orthogonalized U_{IJ} tensor.

	**Atom**	**Wyckoff Position**	**x**	**y**	**z**	**U_{eq} ($Å^2×10^3$)**	**Occ.**
**300 K 0 T**	Zn1	2*i*	0.67595(6)	0.3483(6)	0.23114(6)	0.1907(12)	0.15
	Cu1	2*i*	0.67595(6)	0.3483(6)	0.23114(6)	0.1907(12)	0.85
	Cu2	2*i*	0.88534(6)	0.76887(6)	0.34589(5)	0.1852(12)	1
	Cu3	1*e*	0.5	0.5	0	0.1700(14)	1
	Cu4	1*c*	1	0.5	0	0.1605(14)	1
	Cl1	2*i*	0.76654(13)	0.53450(16)	0.56757(10)	0.363(2)	1
	Cl2	2*i*	0.79006(10)	1.00942(10)	0.5001(9)	0.2361(16)	1
	Cl3	2*i*	0.39084(11)	0.74225(10)	0.11869(9)	0.2280(16)	1
	Cl4	2*i*	1.09057(13)	0.23676(11)	-0.04710(9)	0.2415(16)	1
	O1	2*i*	0.9475(3)	0.5403(3)	0.2304(2)	0.146(4)	1
	O2	2*i*	0.6024(3)	0.5535(3)	0.2341(2)	0.152(4)	1
	O3	2*i*	0.3948(3)	0.1643(3)	0.2054(3)	0.302(5)	1
	O4	2*i*	0.7686(4)	0.1474(4)	0.1913(3)	0.290(5)	1
**130 K 0 T**	Zn1	2*i*	0.67316(10)	0.34672(10)	0.72817(10)	0.105(3)	0.15
	Cu1	2*i*	0.67316(10)	0.34672(10)	0.72817(10)	0.105(3)	0.85
	Cu2	2*i*	0.88518(10)	0.76877(10)	0.84707(9)	0.097(3)	1
	Cu4	1*e*	0.5	0.5	0.5	0.088(3)	1
	Cu4	1*c*	1	0.5	0.5	0.083(3)	1
	Cl1	2*i*	0.7706(2)	0.5399(2)	1.06951(18)	0.238(4)	1
	Cl2	2*i*	0.78952(17)	1.01022(16)	0.99806(15)	0.125(3)	1
	Cl3	2*i*	0.60312(17)	0.25376(16)	0.38132(15)	0.124(3)	1
	Cl4	2*i*	0.91081(17)	0.75853(16)	0.54633(15)	0.119(3)	1
	O1	2*i*	0.9473(5)	0.5404(5)	0.7306(4)	0.084(7)	1
	O2	2*i*	0.6017(5)	0.5538(5)	0.7352(4)	0.088(7)	1
	O3	2*i*	0.3911(6)	0.1628(6)	0.7048(6)	0.163(8)	1
	O4	2*i*	0.7681(6)	0.1446(5)	0.6885(6)	0.155(8)	1
**130 K 0.19 T**	Zn1	2*i*	0.67325(11)	0.34662(10)	0.22721(9)	0.0893(18)	0.15
	Cu1	2*i*	0.67325(11)	0.34662(10)	0.22721(9)	0.0893(18)	0.85
	Cu2	2*i*	0.88492(11)	0.76860(10)	0.34730(9)	0.0846(18)	1
	Cu4	1*e*	0.5	0.5	0	0.074(2)	1
	Cu4	1*c*	1	0.5	0	0.070(2)	1
	Cl1	2*i*	0.7715(2)	0.5413(2)	0.56986(16)	0.212(3)	1
	Cl2	2*i*	0.78939(17)	1.01027(15)	0.49775(14)	0.114(2)	1
	Cl3	2*i*	0.39749(17)	0.74626(15)	0.11938(14)	0.109(2)	1
	Cl4	2*i*	1.08884(17)	0.24137(16)	-0.04671(14)	0.107(2)	1
	O1	2*i*	0.9465(5)	0.5396(5)	0.2306(4)	0.074(6)	1
	O2	2*i*	0.6005(5)	0.5537(5)	0.2347(4)	0.085(6)	1
	O3	2*i*	0.3915(6)	0.1623(5)	0.2049(5)	0.158(7)	1
	O4	2*i*	0.7676(6)	0.1458(5)	0.1896(5)	0.136(7)	1

Table S8. Anisotropic displacement parameters ($Å^2 \times 10^3$) from SCXRD. The anisotropic displacement factor exponent takes the form: $-2\pi^2[h^2a^{*2}U_{11}+2hka^*b^*U_{12}+\ldots]$.

	Atom	U_{11}	U_{22}	U_{33}	U_{23}	U_{13}	U_{12}
**300 K 0 T**	Zn1	17.8(2)	17.3(2)	25.9(2)	12.54(18)	7.00(16)	8.78(16)
	Cu1	17.8(2)	17.3(2)	25.9(2)	12.54(18)	7.00(16)	8.78(16)
	Cu2	16.6(2)	15.2(2)	17.4(2)	2.07(17)	1.79(15)	7.79(15)
	Cu3	21.9(3)	21.2(3)	12.6(3)	8.0(2)	3.1(2)	14.1(2)
	Cu4	18.6(3)	20.5(3)	13.4(3)	9.3(2)	7.8(2)	11.2(2)
	Cl1	36.3(4)	69.0(6)	22.1(4)	24.6(4)	13.8(3)	37.1(4)
	Cl2	19.3(3)	19.2(3)	23.3(4)	1.9(3)	1.5(2)	9.8(2)
	Cl3	27.7(4)	21.4(3)	20.5(3)	7.0(3)	3.9(3)	15.5(3)
	Cl4	27.5(4)	28.8(4)	21.4(3)	15.6(3)	9.6(3)	13.3(3)
	O1	15.4(9)	19.0(9)	11.1(9)	7.3(8)	3.9(7)	8.9(7)
	O2	15.8(9)	18.2(9)	13.2(9)	7.5(8)	4.1(7)	9.0(7)
	O3	24.9(11)	27.1(12)	38.4(14)	18.4(11)	11.0(10)	8.3(9)
	O4	28.5(12)	26.5(12)	43.2(15)	23.9(11)	14.1(10)	14.5(10)
**130 K 0 T**	Zn1	5.0(4)	7.3(4)	19.8(4)	8.5(3)	3.5(3)	1.2(3)
	Cu1	5.0(4)	7.3(4)	19.8(4)	8.5(3)	3.5(3)	1.2(3)
	Cu2	4.2(4)	5.9(4)	14.3(4)	2.8(3)	0.5(3)	0.4(3)
	Cu4	7.0(5)	9.2(5)	11.3(5)	6.0(4)	1.1(4)	3.4(4)
	Cu4	5.8(5)	8.7(5)	11.8(5)	6.9(4)	3.7(4)	2.5(4)
	Cl1	21.3(7)	48.9(9)	20.8(7)	22.9(6)	12.8(5)	26.5(6)
	Cl2	5.5(5)	9.1(5)	16.5(6)	2.8(4)	0.2(4)	1.4(4)
	Cl3	8.6(5)	9.5(5)	17.7(6)	5.7(4)	0.8(4)	3.8(4)
	Cl4	9.0(5)	12.4(5)	16.3(6)	9.7(4)	4.9(4)	3.4(4)
	O1	4.2(14)	9.7(15)	11.3(15)	6.0(12)	2.6(11)	2.1(11)
	O2	3.7(14)	8.8(14)	12.0(15)	4.7(12)	1.2(11)	1.7(12)
	O3	10.6(17)	14.5(16)	27(2)	14.9(15)	6.9(15)	3.7(13)
	O4	10.3(17)	11.8(16)	29(2)	15.8(15)	6.0(14)	2.1(13)
**130 K 0.19 T**	Zn1	10.4(3)	5.5(3)	12.9(3)	6.8(3)	3.9(3)	2.8(3)
	Cu1	10.4(3)	5.5(3)	12.9(3)	6.8(3)	3.9(3)	2.8(3)
	Cu2	10.2(3)	4.2(3)	7.8(3)	1.3(2)	1.1(2)	2.2(3)
	Cu4	12.9(5)	7.1(4)	5.0(4)	4.4(3)	2.3(3)	5.3(4)
	Cu4	11.7(5)	6.7(4)	5.1(4)	4.9(3)	4.3(3)	4.0(4)
	Cl1	26.8(6)	42.2(8)	12.9(5)	18.9(5)	11.7(5)	25.5(6)
	Cl2	12.5(5)	6.9(5)	9.9(5)	1.0(4)	0.8(4)	3.4(4)
	Cl3	14.5(5)	7.3(5)	10.3(5)	3.8(4)	1.7(4)	4.8(4)
	Cl4	15.2(5)	10.6(5)	9.0(5)	7.5(4)	4.8(4)	5.2(4)
	O1	8.1(14)	9.0(14)	7.5(14)	6.0(12)	3.1(11)	3.9(12)
	O2	10.7(15	9.9(15)	7.1(14)	6.0(12)	4.2(12)	4.3(13)
	O3	17.2(17)	11.0(16)	19.4(18)	10.4(14)	6.0(12)	3.1(14)
	O4	--	--	--	--	--	--

Table S9. Bond lengths extracted from the SCXRD structures of $Cu_{2.85}Zn_{0.15}Cl_4(OH)_2(H_2O)_2$ collected with GaKα (λ = 1.34139) radiation at several temperatures and applied magnetic fields. The difference between the structures grown with different applied fields and collected at 130 K is shown as $\Delta_{0.19-0\,T}$, with differences greater than the error bars in **bold**.

**Temperature (K)**	**300**	**130**	**130**	**130**
**Applied field (T)**	**0**	**0**	**0.19**	$\Delta_{0.19-0\,T}$
Cu1–Cu2 (Å)	2.9117(9)	2.9111(9)	2.9058(9)	**-0.0053**
Cu1–Cl1 (Å)	2.5787(11)	2.6047(16)	2.6159(14)	**0.0112**
Cu1–O1 (Å)	2.0134(19)	2.018(3)	2.006(3)	**-0.012**
Cu1–O2 (Å)	2.003(2)	1.996(4)	2.000(3)	0.004
Cu1–O3 (Å)	1.971(2)	1.974(4)	1.974(4)	0
Cu1–O4 (Å)	1.971(3)	1.989(4)	1.980(4)	**-0.009**
Cu1–Cl3 (Å)	2.7937(11)	2.7570(14)	2.7575(13)	0.0005
Cu2–Cl2 (Å)	2.2883(10)	2.2812(13)	2.2804(13)	-0.0008
Cu2–Cl2 (Å)	2.2777(9)	2.2725(14)	2.2716(13)	-0.0009
Cu2–Cl4 (Å)	2.5977(10)	2.5975(14)	2.5998(13)	0.0023
Cu2–O1 (Å)	1.990(2)	1.983(4)	1.985(3)	0.002
Cu2–O2 (Å)	2.0028(19)	1.995(3)	1.998(3)	0.003
Cu3–Cl3 (Å)	2.3190(8)	2.3098(12)	2.3062(11)	**-0.0036**
Cu3–O2 (Å)	1.966(2)	1.968(4)	1.962(3)	-0.006
Cu4–Cl3 (Å)	2.6419(9)	2.6710(11)	2.6707(11)	-0.0003
Cu4–Cl4 (Å)	2.4393(9)	2.3944(12)	2.3920(11)	**-0.0024**
Cu4–O1 (Å)	1.9562(19)	1.954(3)	1.958(3)	0.004

Table S10. Bond angles extracted from the SCXRD structures of $Cu_{2.85}Zn_{0.15}Cl_4(OH)_2(H_2O)_2$ collected with GaKα (λ = 1.34139) radiation at several temperatures and applied magnetic fields. The difference between the structures grown with different applied fields and collected at 130 K is shown as $\Delta_{0.19-0\,T}$, with differences greater than the error bars in **bold**.

**Temperature (K)**	**300**	**130**	**130**	**130**
**Applied field (T)**	**0**	**0**	**0.19**	$\Delta_{0.19-0\,T}$
O1–Cu1–Cl1 (°)	89.23(6)	87.89(10)	87.67(10)	**-0.22**
O2–Cu1–Cl1 (°)	89.22(6)	88.35(11)	88.23(10)	-0.12
O1–Cu1–O2 (°)	80.49(8)	80.39(15)	80.72(14)	**0.33**
O3–Cu1–Cl1 (°)	96.69(8)	96.77(13)	96.55(12)	-0.22
O1–Cu1–O3 (°)	171.95(10)	173.03(17)	173.57(15)	**0.54**
O2–Cu1–O3 (°)	94.09(9)	94.52(16)	94.52(15)	0
O3–Cu1–O4 (°)	91.39(10)	91.57(16)	91.25(16)	-0.32
Cl2–Cu2–Cl2 (°)	87.84(3)	88.33(5)	88.36(5)	0.03
Cl2–Cu2–Cl4 (°)	105.17(3)	104.98(5)	104.86(4)	**-0.12**
Cl2–Cu2–Cl4 (°)	100.41(3)	100.39(5)	100.33(5)	-0.06

O1–Cu2–Cl2 (°)	171.85(6)	172.70(10)	172.74(10)	0.04
O1–Cu2–Cl2 (°)	94.92(6)	94.65(11)	94.74(10)	0.09
O1–Cu2–Cl4 (°)	81.91(6)	81.07(11)	81.07(10)	0
O1–Cu2–O2 (°)	81.05(8)	81.26(15)	81.28(14)	0.02
O2–Cu2–Cl2 (°)	95.06(6)	94.76(11)	94.66(11)	-0.1
O2–Cu2–Cl2 (°)	170.66(7)	170.43(12)	170.65(10)	0.22
O2–Cu2–Cl4 (°)	87.42(6)	87.57(11)	87.47(10)	-0.1
Cl3–Cu3–Cl3 (°)	180	180	180	0
O2–Cu3–Cl3 (°)	90.94(6)	91.07(11)	90.75(10)	**-0.32**
O2–Cu3–Cl3 (°)	89.06(6)	88.93(11)	89.26(10)	**0.33**
O2–Cu3–O2 (°)	180	180	180	0
Cl3–Cu4–Cl3 (°)	180.00(4)	180	180.00(6)	0
Cl4–Cu4–Cl3 (°)	86.82(3)	86.92(4)	86.88(4)	-0.04
Cl4–Cu4–Cl3 (°)	93.18(3)	93.08(4)	93.12(4)	0.04
Cl4–Cu4–Cl4 (°)	180	180.00(2)	180	0
O1–Cu4–Cl3 (°)	85.68(6)	85.07(10)	85.03(10)	-0.04
O1–Cu4–Cl3 (°)	94.32(6)	94.93(10)	94.97(10)	0.04
O1–Cu4–Cl4 (°)	93.14(6)	92.88(11)	92.82(10)	-0.06
O1–Cu4–Cl4 (°)	86.86(6)	87.12(11)	87.18(10)	0.06
O1–Cu4–O1 (°)	180	180	180	0
Cu1–O1–Cu2 (°)	93.31(8)	93.34(15)	93.46(14)	0.12
Cu1–O1–Cu4 (°)	115.62(9)	115.24(17)	115.24(16)	0
Cu2–O1–Cu4 (°)	110.73(10)	110.71(18)	110.56(15)	-0.15
Cu1–O2–Cu2 (°)	93.25(9)	93.67(16)	93.24(15)	**-0.43**
Cu1–O2–Cu3 (°)	113.27(9)	112.53(16)	112.37(16)	-0.16
Cu2–O2–Cu3 (°)	114.43(9)	114.03(16)	114.06(16)	0.03

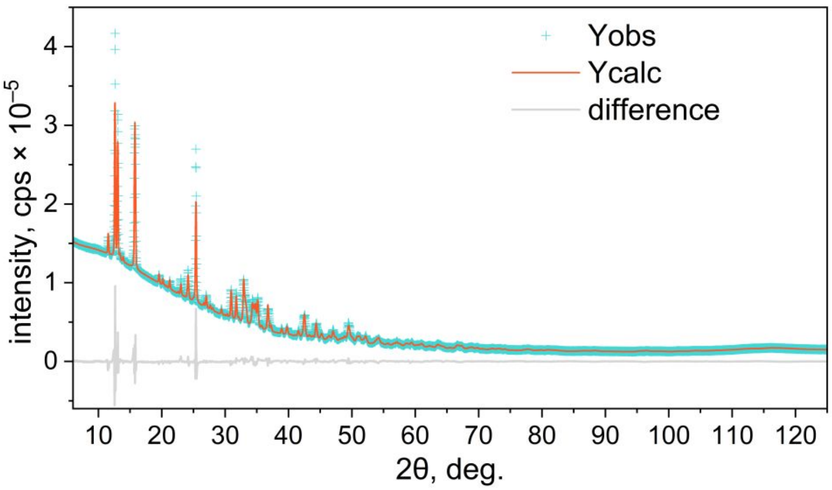

Figure S9. Rietveld refinement in space group *P*-1 of PXRD data from a few $(Cu,Zn)_3Cl_4(OH)_2{\cdot}2H_2O$ crystals. Note: the mismatch between observed and calculated intensities can be explained by the small amount of sample (< 1 mg) and insufficient grinding.

Table S11. Parameters extracted from a Rietveld refinement of laboratory PXRD data of $(Cu,Zn)_3Cl_4(OH)_2{\cdot}2H_2O$ in space group *P*-1 measured at room temperature.

a **(Å)**	7.382(4)
b **(Å)**	8.445(5)
c **(Å)**	8.735(5)
**α (°)**	115.995(4)
**β (°)**	90.148(5)
**γ (°)**	113.620(5)
**V (Å^3)**	437.8(4)
**r_exp**	0.49
**r_exp_dash**	2.93
**r_wp**	3.40
**r_wp_dash**	20.46
**r_p**	1.78
**r_p_dash**	18.90
**Weighted Durbin Watson**	0.38
**gof**	6.98
**Preferred Orientation**	(0 1 -1), (1 -2 2)

Table S12. Fractional atomic coordinates and equivalent isotropic displacement parameters extracted from a Rietveld refinement of laboratory PXRD data of $(Cu,Zn)_3Cl_4(OH)_2{\cdot}2H_2O$ in space group *P*-1 measured at room temperature.

Site	Wyckoff Position	x	y	z	occ	B_{eq}
Cu1	2*i*	0.670(1)	0.350(1)	0.240(1)	1	1.3(3)
Cu2	2*i*	0.890(2)	0.767(1)	0.340(1)	1	2.1(3)
Cu3	1*e*	0.5	0.5	0	1	1.5(2)
Cu4	1*c*	1	0.5	0	1	1.4(4)
Cl1	2*i*	0.759(2)	0.534(2)	0.560(2)	1	0.6(3)
Cl2	2*i*	0.800(2)	1.005(2)	0.492(2)	1	2.6(4)
Cl3	2*i*	0.380(3)	0.740(2)	0.120(2)	1	3.2(5)
Cl4	2*i*	1.099(2)	0.239(2)	-0.040(2)	1	3.2(5)
O1	2*i*	0.947(4)	0.530(4)	0.225(3)	1	1.4(9)
O2	2*i*	0.592(5)	0.550(5)	0.230(4)	1	2.2(10)
O3	2*i*	0.395(4)	0.170(4)	0.210(3)	1	0.4(9)
O4	2*i*	0.770(5)	0.150(4)	0.198(4)	1	0.5(8)

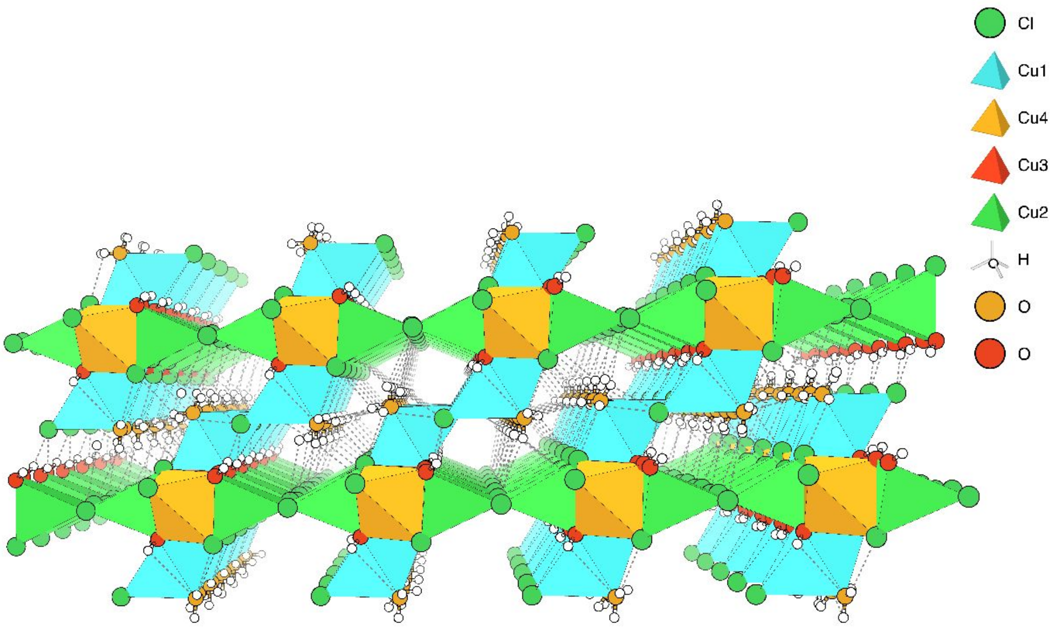

Figure S10. The H···O and H···Cl hydrogen bonding between the $(Cu,Zn)_3Cl_4(OH)_2{\cdot}2H_2O$ layers in $(Cu,Zn)_3Cl_4(OH)_2{\cdot}2H_2O$ structure. White, red, green, blue, and orange spheres represent H, O, Cl, Cu atoms, and O atom in water molecules, respectively. Blue, green, red, and orange polyhedra represent Cu1, Cu2, Cu3, and Cu4, respectively. Zn atoms in the structure are omitted for clarity. The gray dashed lines are hydrogen bonding.

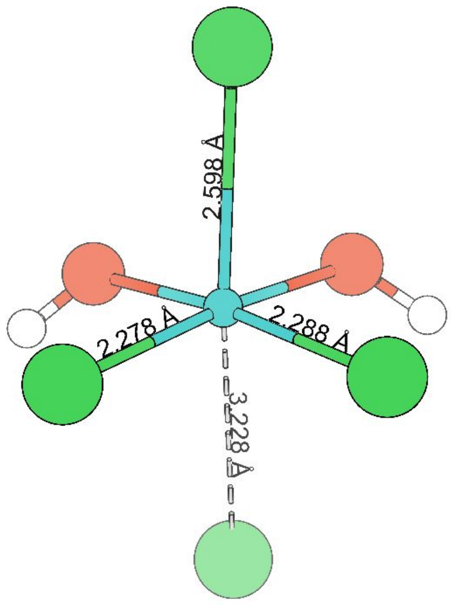

Figure S11. Cu2 coordination environment. White, red, green, and blue spheres represent H, O, Cl, and Cu atoms, respectively. The gray dashed line is the ionic interaction between Cu2 and Cl1 which serves as additional bonding moiety between the $(Cu,Zn)_3Cl_4(OH)_2{\cdot}2H_2O$ layers.

Torsion angle

The $(Cu,Zn)_3Cl_4(OH)_2{\cdot}2H_2O$ crystal structure has layers of Cu/Zn bowties which are staggered at an angle. For the crystal formed under no magnetic field, the torsion angles are calculated as 148.59° for the side angle and 152.54° for the top angle, as shown in Figure S12. For the crystal formed under the 0.19 T magnetic field, the torsion angle is 148.40° for the side and 152.80° for the top.

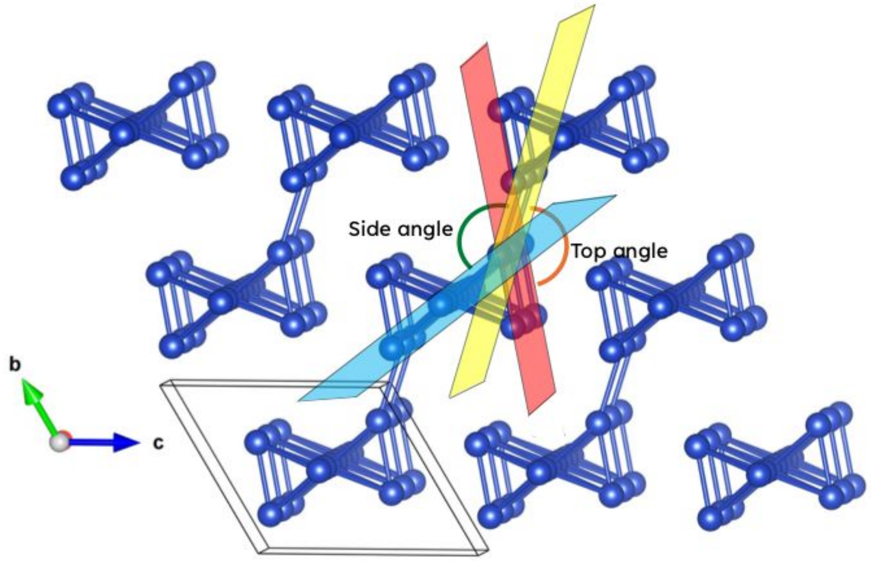

Figure S12: Depiction of the torsion angles of the Cu/Zn sublattice.

c. $CuCl_2{\cdot}2H_2O$

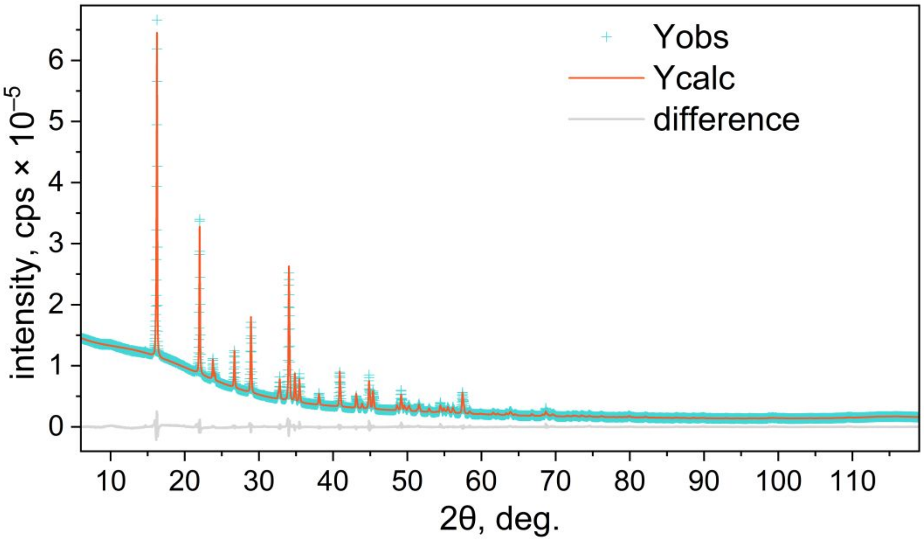

Figure S13. Rietveld refinement of $CuCl_2 \cdot 2H_2O$_0T laboratory PXRD data in space group *Pmna*.

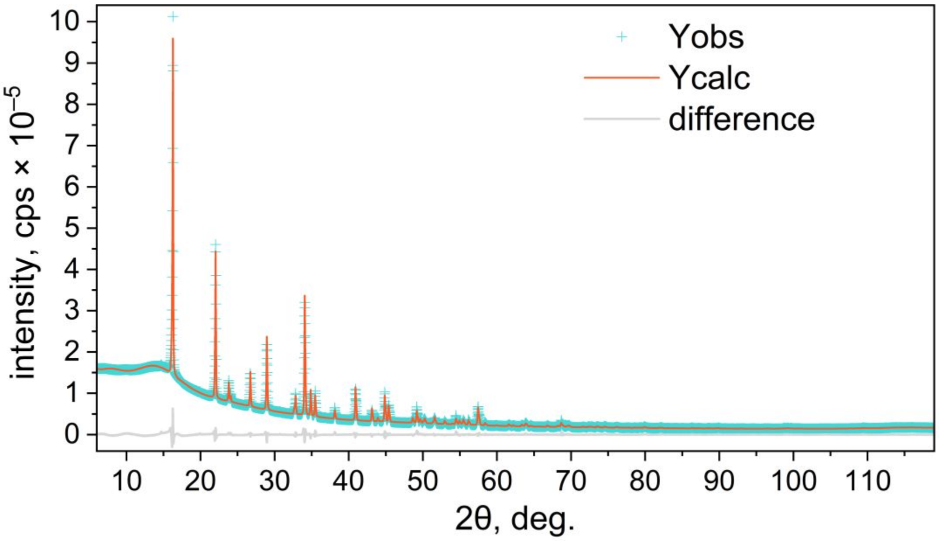

Figure S14. Rietveld refinement of $CuCl_2 \cdot 2H_2O$_0.19T laboratory PXRD data in space group *Pmna*.

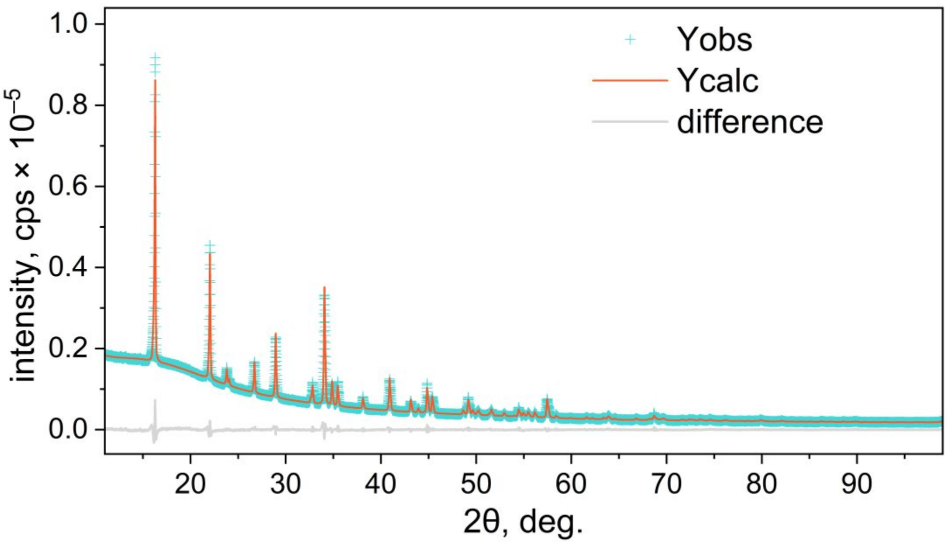

Figure S15. Rietveld refinement of $CuCl_2·2H_2O$_0.37T laboratory PXRD data in space group *Pmna*.

Table S13. Parameters extracted from Rietveld refinements of laboratory PXRD data of $CuCl_2·2H_2O$ in space group *Pmna* synthesized under various magnetic fields and measured at room temperature. The differences between the structures grown with different applied fields are shown as $\Delta_{0.19-0\,T}$ and $\Delta_{0.37-0\,T}$, with differences greater than the sum of the error bars in **bold**.

	0 T	0.19 T	0.37 T	$\Delta_{0.19-0\,T}$	$\Delta_{0.37-0\,T}$
***a* (Å)**	8.0917(3)	8.0930(2)	8.0929(2)	**0.0013**	**0.0012**
***b* (Å)**	3.7476(1)	3.7479(1)	3.7480(1)	**0.0003**	**0.0004**
***c* (Å)**	7.4175(3)	7.4183(2)	7.4186(2)	**0.0008**	**0.0011**
**V (Å^3)**	224.928(15)	225.007(12)	225.025(12)	**0.079**	**0.097**
**r_exp**	0.49	0.46	1.29		
**r_exp_dash**	2.68	2.20	6.58		
**r_wp**	2.22	2.59	2.60		
**r_wp_dash**	12.21	12.45	13.26		
**r_p**	1.45	1.72	1.76		
**r_p_dash**	19.03	19.08	19.60		
**Weighted Durbin Watson**	0.28	0.26	0.30		
**gof**	4.56	5.66	2.01		
**Preferred Orientation**	(1 0 1), (2 0 0)	(1 0 1), (2 0 0)	(1 0 1), (2 0 0)		

Table S14. Fractional atomic coordinates and equivalent isotropic displacement parameters from Rietveld refinements of laboratory PXRD data of $CuCl_2·2H_2O$ in space group *Pmna* synthesized under various magnetic fields and measured at room temperature.

	**Atom**	**Wyckoff Position**	**x**	**y**	**z**	**B$_{eq}$**	**Occ.**
**0 T**	Cu1	2*a*	0	0.5	0.5	4.61(7)	1
	Cl1	4*h*	0	0.1205(4)	0.2590(2)	4.55(7)	1
	O1	4*e*	0.2450(4)	0.5	0.5	3.7(1)	1
**0.19 T**	Cu1	2*a*	0	0.5	0.5	5.12(7)	1
	Cl1	4*h*	0	0.1197(4)	0.2603(3)	5.44(8)	1
	O1	4*e*	0.2420(4)	0.5	0.5	5.2(1)	1
**0.37 T**	Cu1	2*a*	0	0.5	0.5	4.26(6)	1
	Cl1	4*h*	0	0.1211(4)	0.2589(2)	4.31(6)	1
	O1	4*e*	0.2442(4)	0.5	0.5	3.98(9)	1

d. Atacamite $Cu_2(OH)_3Cl$

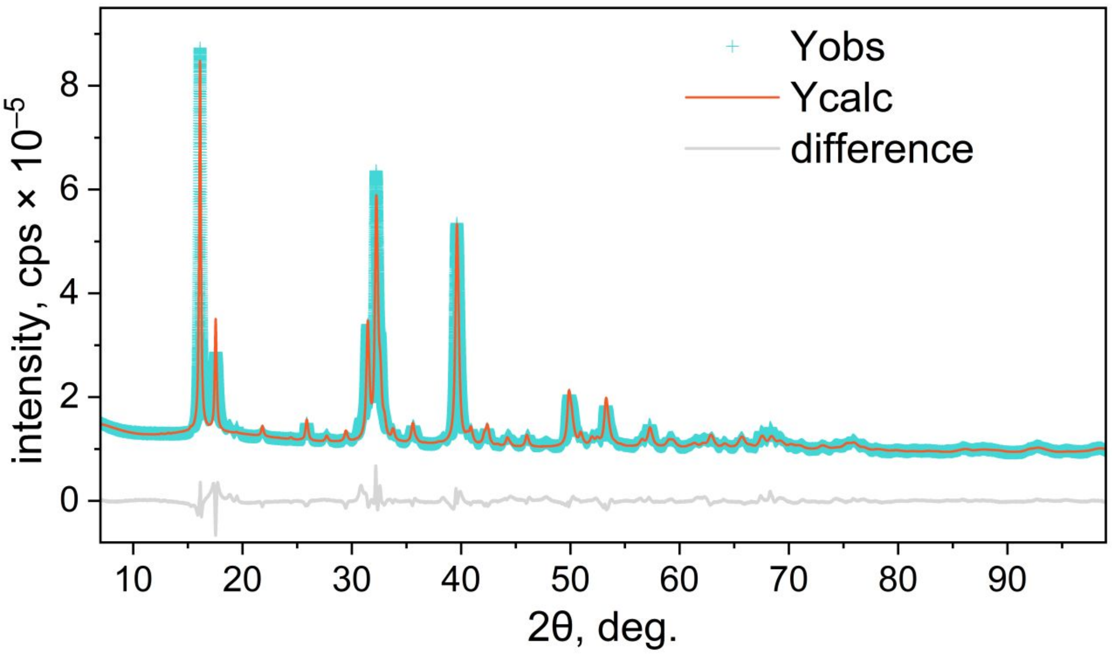

Figure S16. Rietveld refinement of atacamite $Cu_2(OH)_3Cl$_0T laboratory PXRD data in space group *Pmna*.

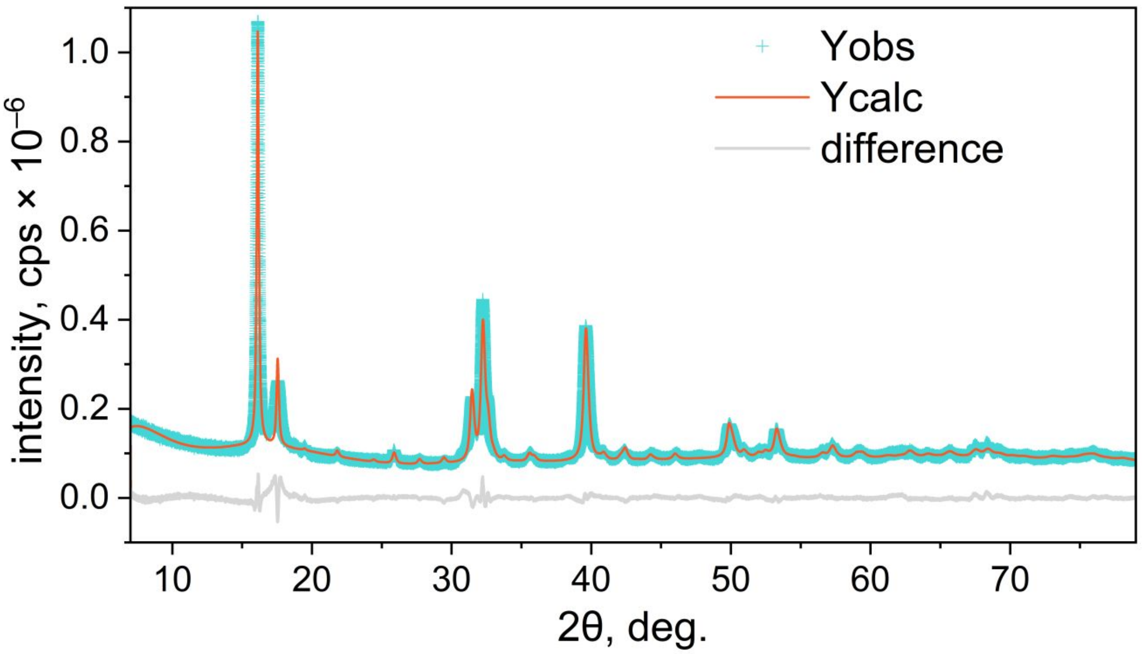

Figure S17. Rietveld refinement of atacamite $Cu_2(OH)_3Cl$_0.19T laboratory PXRD data in space group *Pmna*.

Table S15. Parameters extracted from Rietveld refinements of PXRD data of atacamite $Cu_2(OH)_3Cl$ in space group *Pnma* synthesized under various magnetic fields and measured at room temperature. The difference between the structures grown with different applied fields is shown as $\Delta_{\mathbf{0.19-0\,T}}$, with differences greater than the error bars in **bold**.

	**0 T**	**0.19 T**	$\Delta_{0.19-0\,T}$
a **(Å)**	6.0510(12)	6.0549(11)	**0.0039**
b **(Å)**	6.8697(13)	6.8689(13)	-0.0008
c **(Å)**	9.0944(18)	9.0916(17)	-0.0028
**V (Å^3)**	378.04(13)	378.12(12)	0.08
**r_exp**	0.88	0.30	
**r_exp_dash**	3.51	1.17	
**r_wp**	4.12	4.63	
**r_wp_dash**	16.48	17.90	
**r_p**	2.77	3.18	
**r_p_dash**	18.96	21.00	
**Weighted Durbin Watson**	0.001	0.008	
**gof**	4.70	15.27	
**Preferred Orientation**	(0 1 1), (0 0 2)	(0 1 1), (0 0 2)	

Table S16. Fractional atomic coordinates and equivalent isotropic displacement parameters from Rietveld refinements of PXRD data of atacamite $Cu_2(OH)_3Cl$ in space group *Pnma* synthesized under various magnetic fields and measured at room temperature.

	**Atom**	**Wyckoff Position**	**x**	**y**	**z**	$\mathbf{B_{eq}}$	**Occ.**
**0 T**	Cu1	4*a*	0	0	0	1.04(3)	1
	Cu2	4*c*	0.1825 (2)	0.25	0.2569(2)	1.04(3)	1
	Cl1	4*c*	0.3489(4)	0.75	0.0650(4)	4.78(6)	1
	O1	4*c*	0.1600(7)	0.25	-0.0100(8)	4.78(6)	1
	O2	8*d*	0.4600(6)	0.0550(4)	0.2738(4)	4.78(6)	1
**0.19 T**	Cu1	4*a*	0	0	0	1.73(4)	1
	Cu2	4*c*	0.1884(3)	0.25	0.2479 (3)	1.73(4)	1
	Cl1	4*c*	0.3400(4)	0.75	0.0586 (4)	2.90(6)	1
	O1	4*c*	0.1523(8)	0.25	-0.0100(10)	2.90(6)	1
	O2	8*d*	0.4600(7)	0.0550(5)	0.2875(5)	2.90(6)	1

5. ADDITIONAL MAGNETIC DATA

a. Herbertsmithite

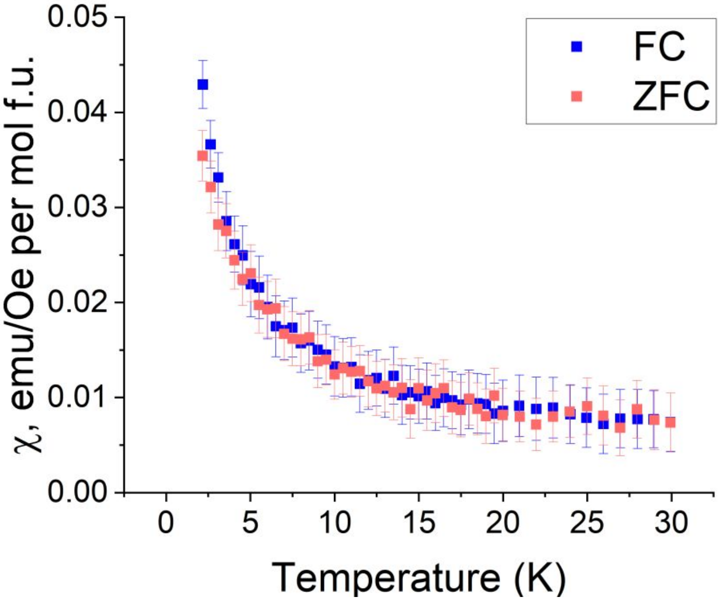

Figure S18. DC magnetization for HBS_0T collected at 0.005 T. Orange and blue represent ZFC and FC data, respectively.

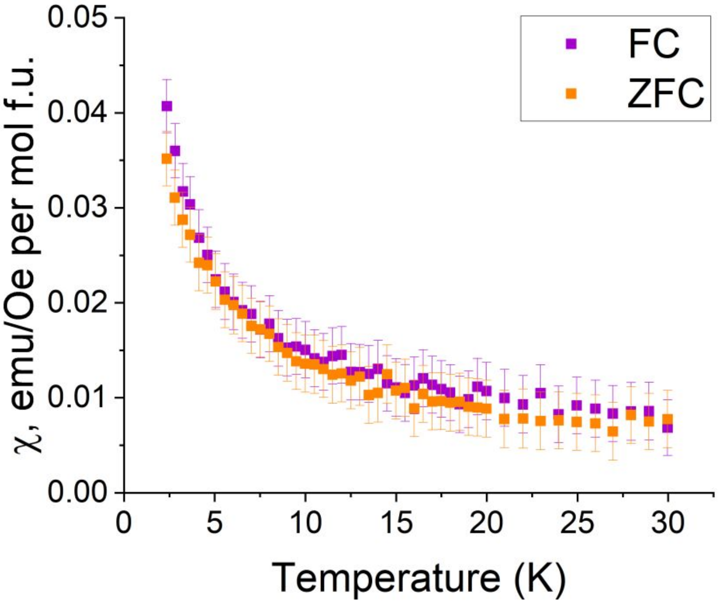

Figure S19. DC magnetization for HBS_0.09T collected at 0.005 T. Orange and purple represent ZFC and FC data, respectively.

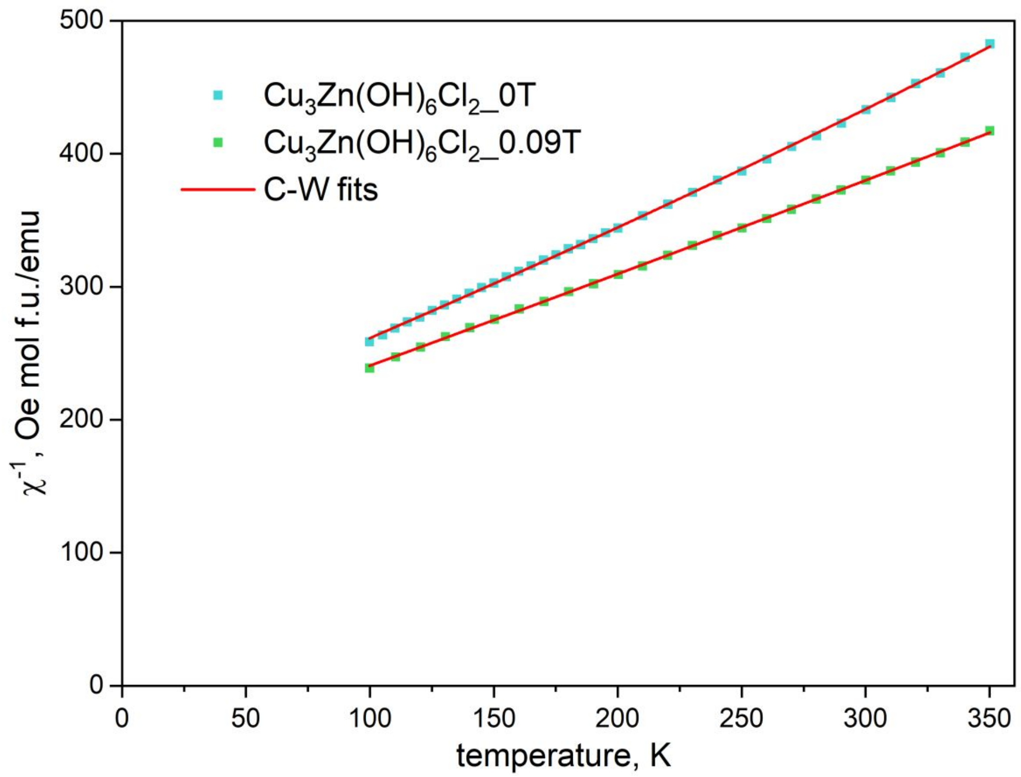

Figure S20. Inverse DC susceptibility data of HBS synthesized under 0 T and 0.09 T. The data were collected under an applied field of 1 T.

Table S17. Curie Weiss fits for HBS synthesized under varying magnetic fields.

**sample**	**HBS_0T**	**HBS_0.09T**	**literature**
**Θ, K**	–263(10)	–265(10)	-314;[2] -300(20)[3]
**χ_0**	–0.00045(7)	–0.00015(7)	–
**$\mu_{eff}(Cu^{2+})$, μ_B**	2.0(1)	2.0(1)	–
**R^2**	0.99974	0.99981	–

b. $(Cu,Zn)_3Cl_4(OH)_2{\cdot}2H_2O$

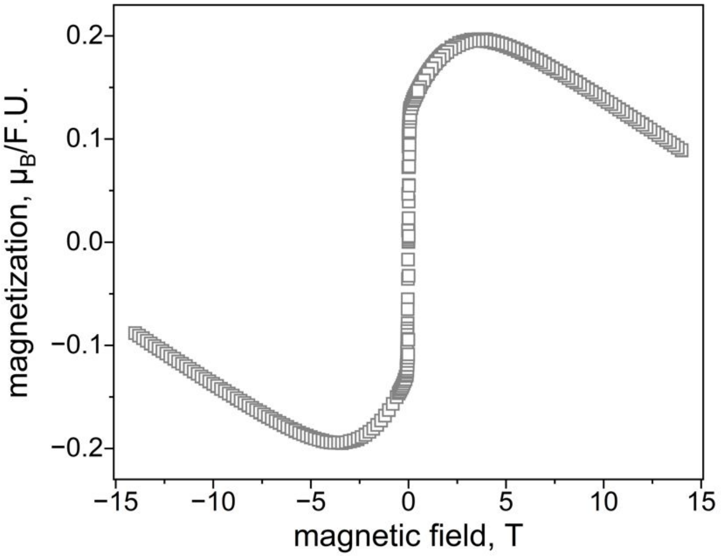

Figure S21. Magnetization (M) as a function of applied magnetic field at 2 K for a few $(Cu,Zn)_3Cl_4(OH)_2{\cdot}2H_2O$ crystals. Note: there was a large uncertainty in the sample mass, so the magnetization values may not be quantitative; the error bars in the plot are smaller than the square size.

c. $CuCl_2{\cdot}2H_2O$

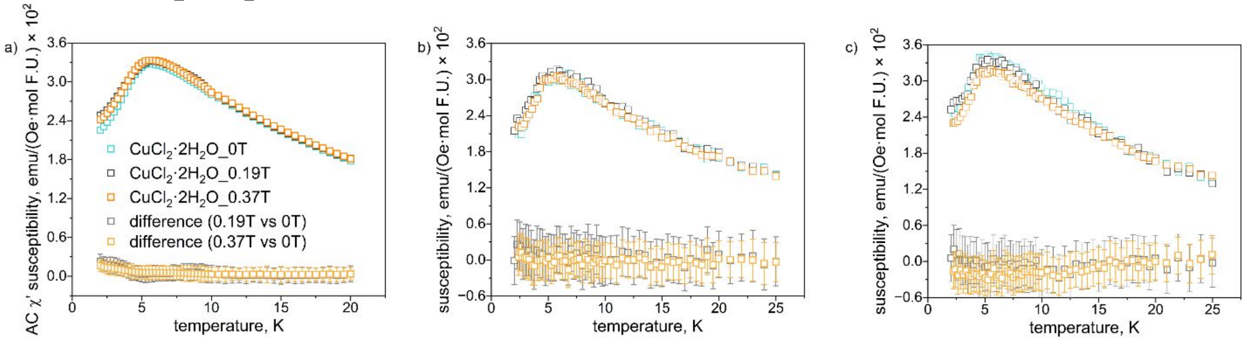

Figure S22. (*a*) Real part of the AC magnetic susceptibility collected at 0 T, (*b*) ZFC and (*c*) FC DC magnetization (collected at 0.005 T) for $CuCl_2{\cdot}2H_2O$ synthesized under various magnetic field strengths: 0 T (blue), 0.19 T (gray), and 0.37 T (orange), respectively. For (*a-c*), the difference between $CuCl_2{\cdot}2H_2O$_0.19T (gray), $CuCl_2{\cdot}2H_2O$_0.37T (orange), and $CuCl_2{\cdot}2H_2O$_0T samples is shown with error bars.

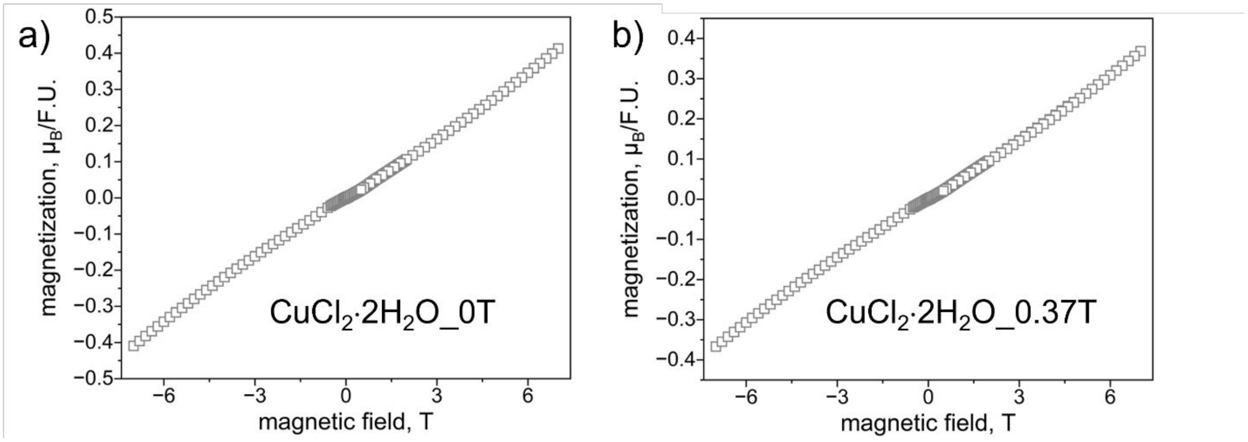

Figure S23. Magnetization (M) as a function of applied magnetic field at 2 K for (a) $CuCl_2·2H_2O$_0T and (b) $CuCl_2·2H_2O$_0.37T powders. Note: the error bars in the plot are smaller than the square size.

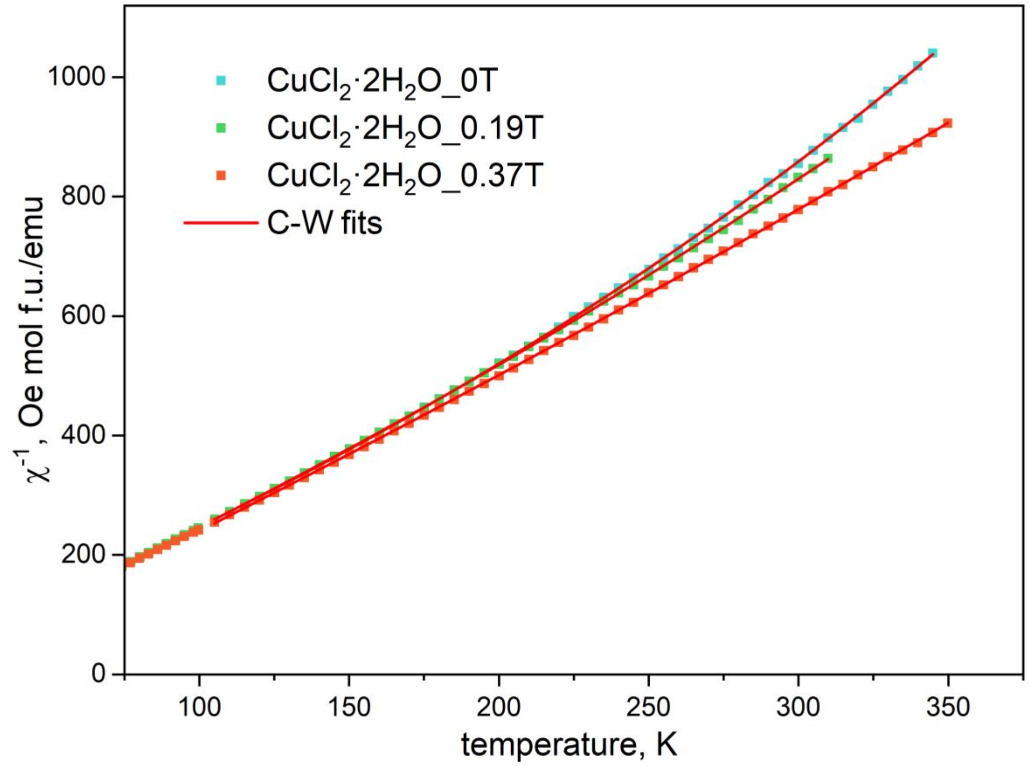

Figure S24. Inverse DC susceptibility data of for $CuCl_2·2H_2O$ synthesized under 0 T, 0.19 T, and 0.37T. The data were collected under an applied field of 1 T.

Table S18. AFM peak and Curie Weiss fits for $CuCl_2 \cdot 2H_2O$ synthesized under varying magnetic fields.

sample	$CuCl_2 \cdot 2H_2O$_0T	$CuCl_2 \cdot 2H_2O$_0.19T	$CuCl_2 \cdot 2H_2O$_0.37T	literature
**T_N, K AC**	5.7	5.7	5.7	4.3[4]<br>5.5[5]
**T_N, K DC ZFC**	5.8	5.9	5.7	
**T_N, K DC FC**	5.5	5.7	5.6	
**Θ, K**	−10.5(9)	−3.0(9)	0.0(6)	–5.3<br>–4.7(3)
**χ_0**	−0.000430(9)	−0.000264(9)	−0.000148(6)	0.0328(4)[5]
**$\mu_{eff}(Cu^{2+})$, μ_B**	1.990(8)	1.887(9)	1.852(6),	1.909(6)
**R^2**	0.99996	0.99995	0.99998	–

Table S19. Analysis of the Curie Weiss fit stability for $CuCl_2 \cdot 2H_2O$ synthesized under varying magnetic fields.

**sample**	**$CuCl_2 \cdot 2H_2O$_0T**	**$CuCl_2 \cdot 2H_2O$_0.19T**	**$CuCl_2 \cdot 2H_2O$_0.37T**
temperature range	**Θ, K; χ_0 (free); $\mu_{eff}(Cu^{2+})$, μ_B; R^2**		
100–310 K	−10.5(9); −0.000430(9); 1.990(8), 0.99996	−3.0(9); −0.000264(9); 1.887(9), 0.99995	0.0(6); −0.000148(6); 1.852(6), 0.99998
150–310 K	−12(2); −0.00044(2); 2.01(2), 0.99993	−4(2); −0.00027(2); 1.90(2), 0.99989	3(1); −0.00012(1); 1.83(1), 0.99996
200–310 K	−11(7); −0.00044(5); 1.99(5), 0.99982	−25(8); −0.00041(5); 2.04(6), 0.99979	−6(2); −0.00069(2); 1.75(3), 0.99993
temperature range	**Θ, K; χ_0(fixed at $\chi_0$for 100–310 K refinement); $\mu_{eff}(Cu^{2+})$, μ_B; R^2**		
150–310 K	−10.6(4); −0.000430; 1.990(1), 0.99993	−3.1(4); −0.000264; 1.888(2), 0.99989	0.4(3); −0.000148; 1.851(1), 0.99996
200–310 K	−10.2(8); −0.000430; 1.989(3), 0.99983	−3.6(9); −0.000264; 1.889(3), 0.99972	0.0(6); −0.000148; 1.853(2), 0.99989

d. Atacamite $Cu_2(OH)_3Cl$

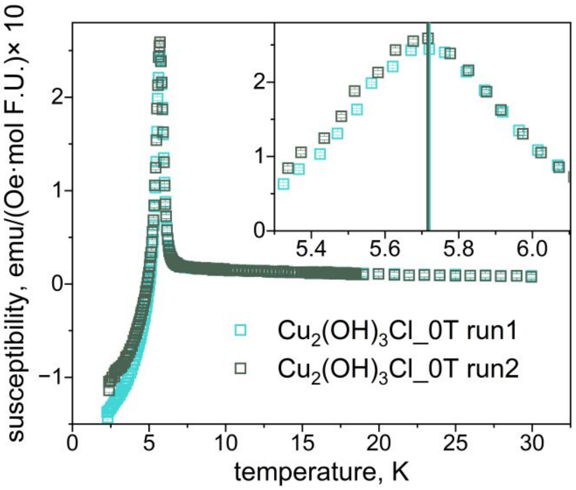

Figure S25. Two different measurement runs of ZFC susceptibility (measured at 0.005 T) for the same atacamite $Cu_2(OH)_3Cl$_0T sample synthesized under zero field. The inset demonstrates that T_N for $Cu_2(OH)_3Cl$_0T is independent of sample loading in the PPMS.

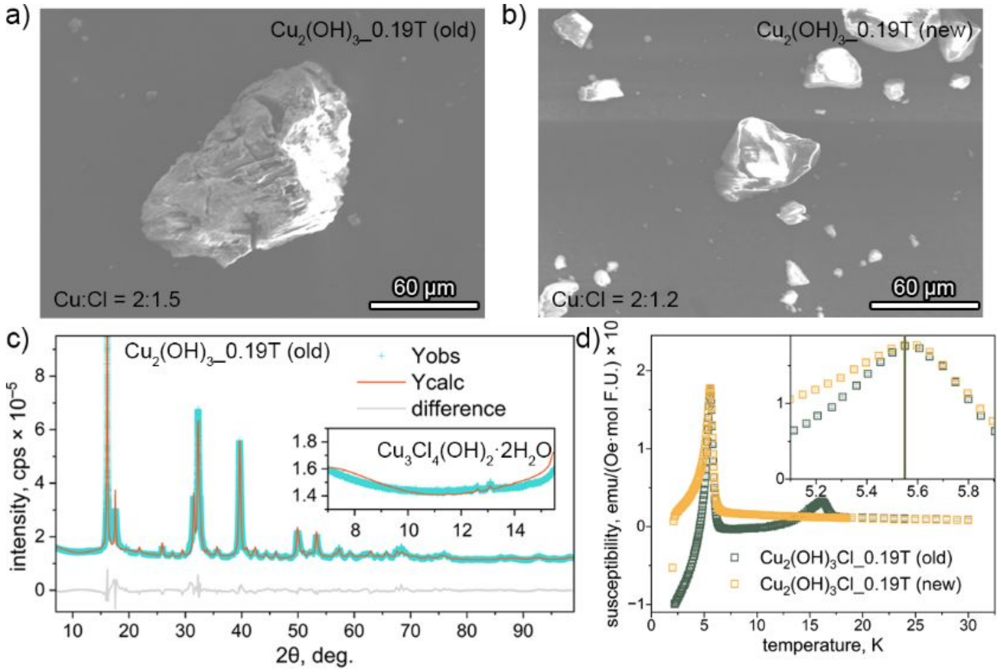

Figure S26. Comparison of $Cu_2(OH)_3Cl$_0.19T aliquots taken from the same batch and washed with acetone two months apart. SEM images of (*a*) $Cu_2(OH)_3Cl$_0.19T (old) and (*b*) $Cu_2(OH)_3Cl$_0.19T (new) powder with the EDS results inset. (*c*) Rietveld refinement of laboratory PXRD data of the $Cu_2(OH)_3Cl$_0.19T (old) sample. The inset shows a $Cu_3Cl_4(OH)_2·2H_2O$ impurity identified at 0.3 wt.% level. (*d*) ZFC susceptibility (measured at 0.005 T) for $Cu_2(OH)_3Cl$_0.19T

(old) and $Cu_2(OH)_3Cl$_0.19T (new). The inset demonstrates that T_N for $Cu_2(OH)_3Cl$_0.19T is independent of the $Cu_2(OH)_3Cl$ particle size or impurity presence.

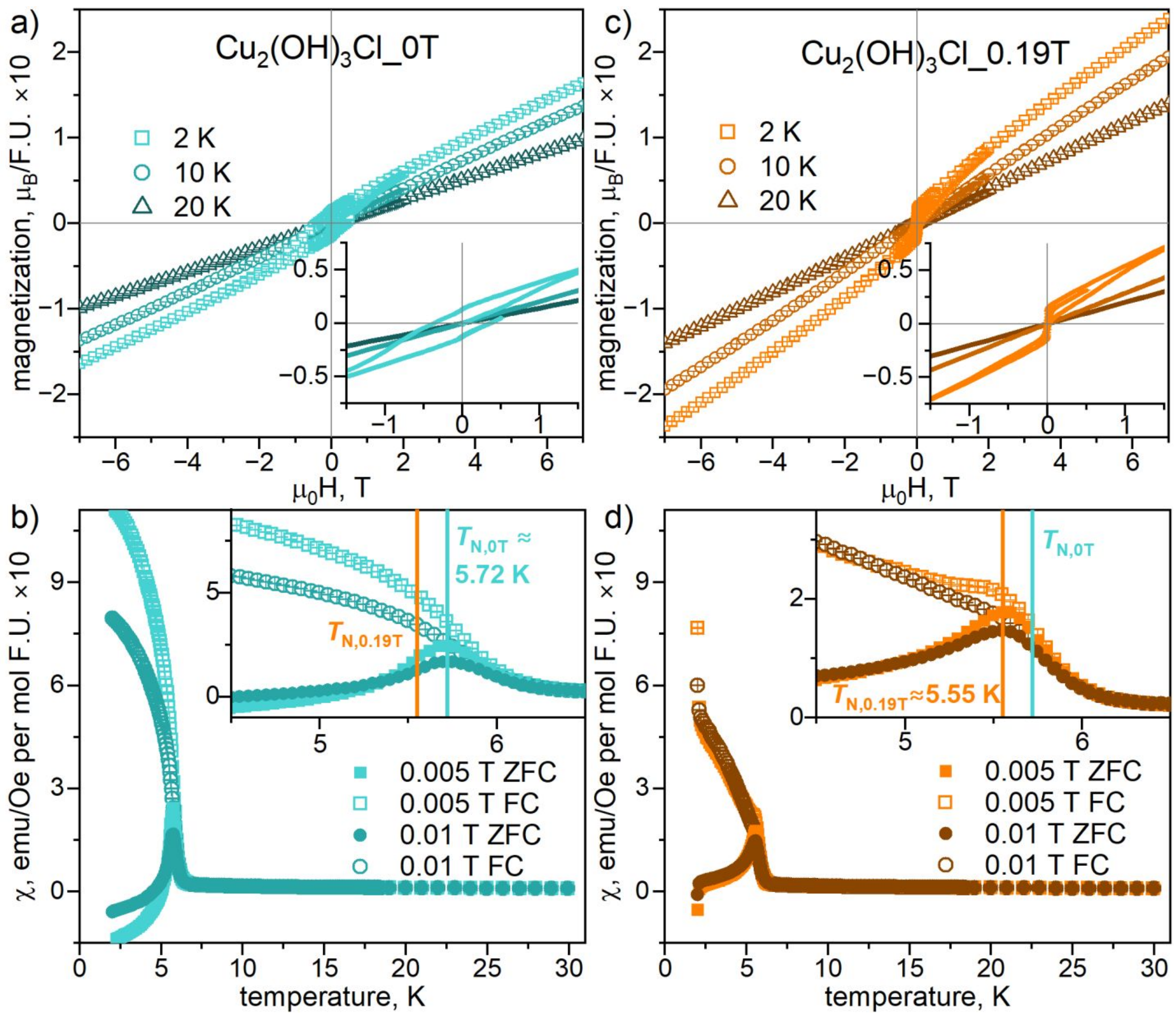

Figure S27. DC magnetization data for atacamite $Cu_2(OH)_3Cl$ synthesized under 0 T (a,b) and 0.19 T (c,d). a,c) Magnetization (M) as a function of applied magnetic field at several temperatures for a) $Cu_2(OH)_3Cl$_0T and c) $Cu_2(OH)_3Cl$_0.19T. Note: the error bars in the plot are smaller than the symbol size. b,d) ZFC and FC DC susceptibility data for b) $Cu_2(OH)_3Cl$_0T and d) $Cu_2(OH)_3Cl$_0.19T collected under several applied fields. Closed and open symbols represent ZFC and FC data, respectively.

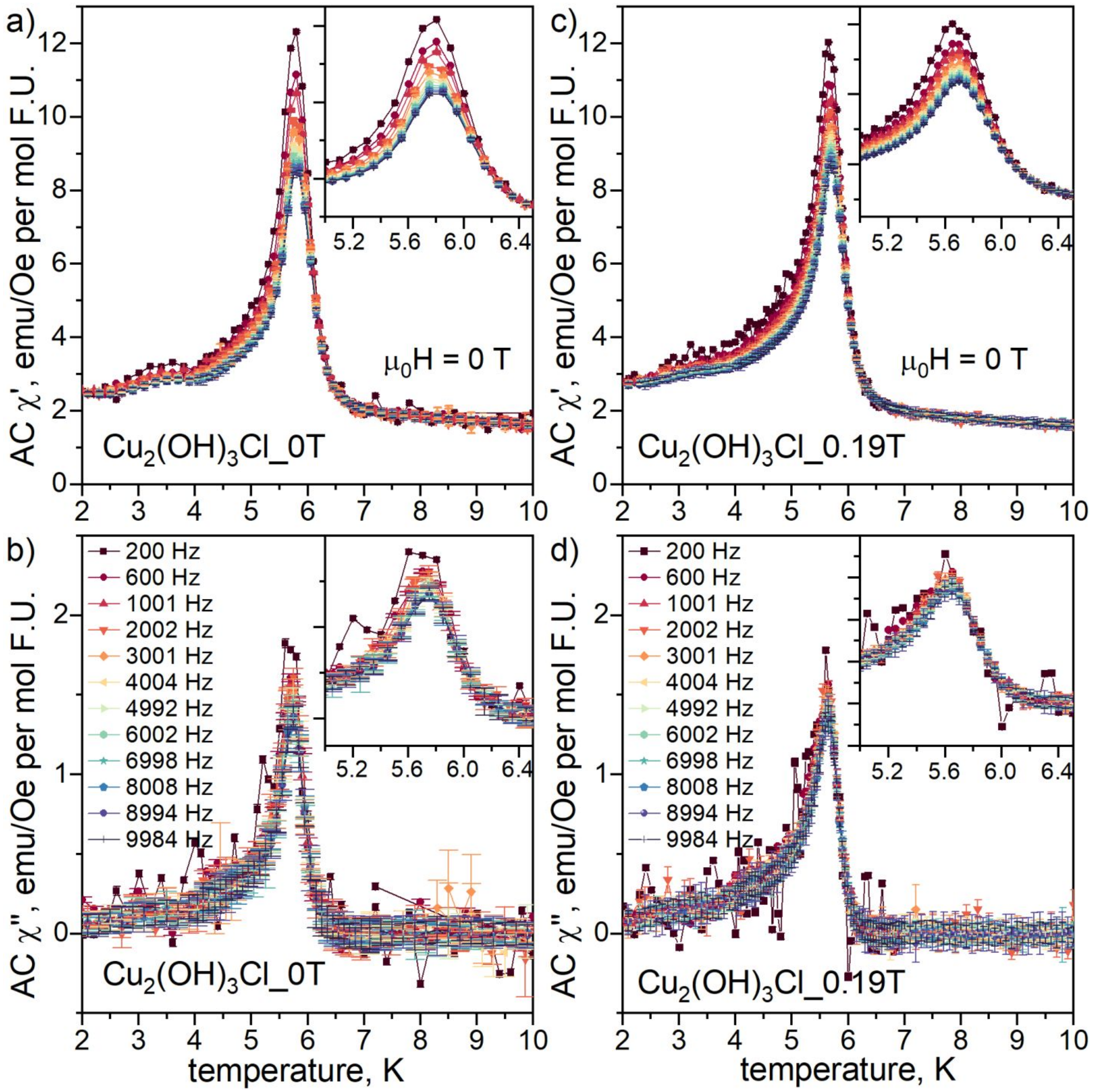

Figure S28. Frequency dependence of the a,c) real (χ’) and b,d) imaginary (χ’’) parts of the AC susceptibility data for atacamite $Cu_2(OH)_3Cl$ synthesized under 0 T (a,b) and 0.19 T (c,d). The data were collected under an applied field of 0 T.

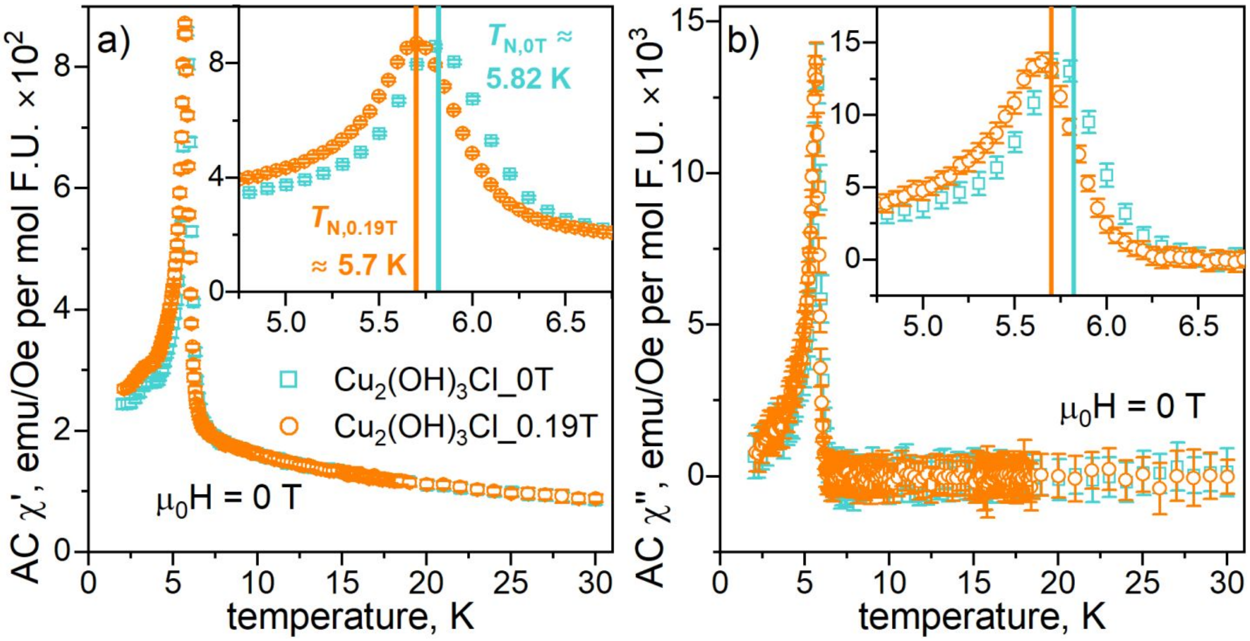

Figure S29. a) Real (χ') and b) imaginary (χ'') parts of the AC susceptibility data for atacamite $Cu_2(OH)_3Cl$ synthesized under 0 T and 0.19 T. The data were collected under an applied field of 0 T and at a frequency of 9984 Hz.

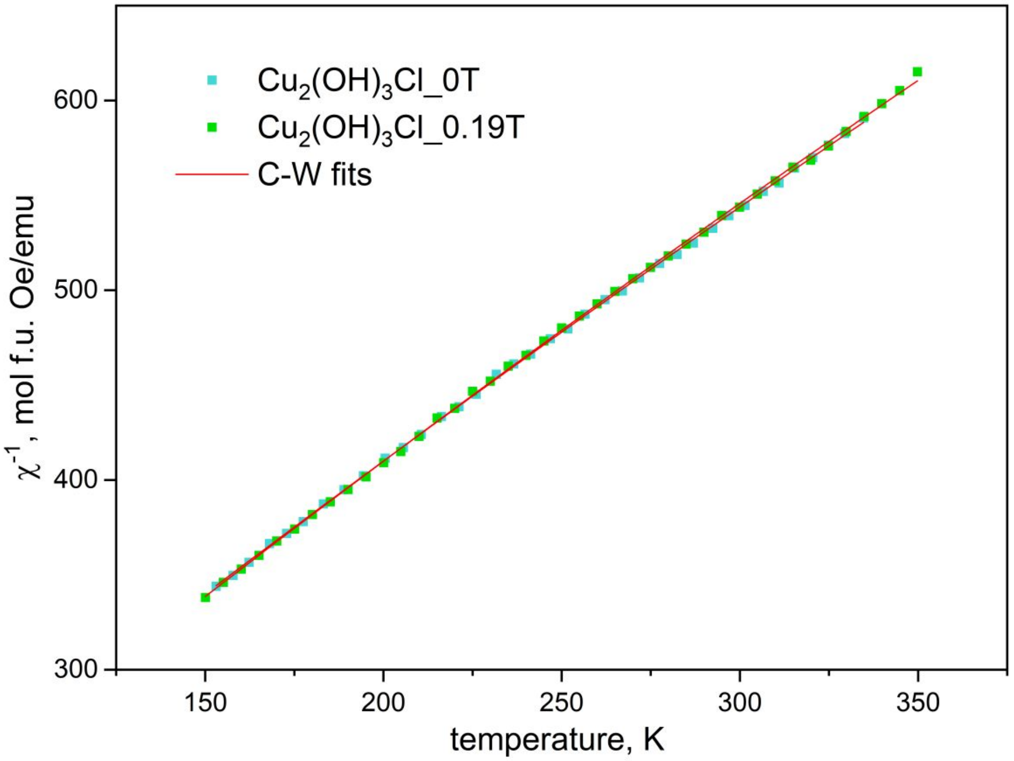

Figure S30. Inverse DC susceptibility data of for atacamite $Cu_2(OH)_3Cl$ synthesized under 0 T and 0.19 T. The data were collected under an applied field of 1 T.

Table S20. Néel temperatures and parameters extracted from Curie Weiss fits for atacamite $Cu_2(OH)_3Cl$ synthesized under varying magnetic fields.

sample	$Cu_2(OH)_3Cl$_0T	$Cu_2(OH)_3Cl$_0.19T	literature
T_N, K AC	5.82	5.70	5.8 (synthetic sample)[6]
T_N, K DC ZFC	5.72	5.55	5.3 (synthetic sample)[6]*<br>8.4 – 9.0 (natural samples)[7,8]
Θ, K	−73(5)	−68(3)	−93 (synthetic sample)[6]*<br>−103 – −136 (natural samples)[7,8]
χ_0	0.00020(3)	0.00021(2)	–
$\mu_{eff}(Cu^{2+})$, μ_B	1.56(3)	1.59(4)	1.79 (synthetic sample)[6]*
R^2	0.99979	0.99973	–

*These DC data from literature were collected via the Faraday method with a magnetic balance and thus are not necessarily directly comparable to the data presented here.

Table S21. Analysis of the Curie Weiss fit stability for atacamite $Cu_2(OH)_3Cl$ synthesized under varying magnetic fields.

sample	$Cu_2(OH)_3Cl$_0T	$Cu_2(OH)_3Cl$_0.19T
temperature range	**Θ, K; χ_0 (free); $\mu_{eff}(Cu^{2+})$, μ_B; R^2**	
150–350 K	−73(5); 0.00020(3);<br>1.56(3), 0.99979	−67(3); 0.00021(2);<br>1.54(1), 0.99973
200–150 K	−99(15); 0.00006(8);<br>1.68(7), 0.99958	−78(8); 0.00016(4);<br>1.59(4), 0.99942
250–350 K	−293(82); −0.0008(4);<br>2.5(3), 0.99951	−324(44); −0.0009(2);<br>2.6(2), 0.99942
temperature range	**Θ, K; χ_0 (fixed at 0); $\mu_{eff}(Cu^{2+})$, μ_B; R^2**	
150–350 K	−105(1); 0;<br>1.725(3), 0.99958	−101.2(7); 0;<br>1.715(2), 0.99946
200–150 K	−112(2); 0;<br>1.741(4), 0.99958	−109(1); 0;<br>1.733(2), 0.99937
250–350 K	−112(3); 0;<br>1.742(6), 0.99923	−114(2); 0;<br>1.742(4), 0.99937

REFERENCES

(1) Zagorac, D.; Müller, H.; Ruehl, S.; Zagorac, J.; Rehme, S. Recent Developments in the Inorganic Crystal Structure Database: Theoretical Crystal Structure Data and Related Features. *J. Appl. Crystallogr.* **2019**, *52* (5), 918–925.

(2) Shores, M. P.; Nytko, E. A.; Bartlett, B. M.; Nocera, D. G. A Structurally Perfect $S = {}^{1}/_{2}$ Kagomé Antiferromagnet. *J. Am. Chem. Soc.* **2005**, *127* (39), 13462–13463.

(3) Helton, J. S.; Matan, K.; Shores, M. P.; Nytko, E. A.; Bartlett, B. M.; Yoshida, Y.; Takano, Y.; Suslov, A.; Qiu, Y.; Chung, J.-H.; Nocera, D. G.; Lee, Y. S. Spin Dynamics of the Spin-1/2 Kagome Lattice Antiferromagnet ZnCu3(OH)6Cl2. *Phys. Rev. Lett.* **2007**, *98* (10), 107204.

(4) Schmitt, M.; Janson, O.; Schmidt, M.; Hoffmann, S.; Schnelle, W.; Drechsler, S.-L.; Rosner, H. Crystal-Water-Induced Switching of Magnetically Active Orbitals in CuCl2. *Phys. Rev. B* **2009**, *79* (24), 245119.

(5) DeFotis, G. C.; Hampton, A. S.; Van Dongen, M. J.; Komatsu, C. H.; Benday, N. S.; Davis, C. M.; Hays, K.; Wagner, M. J. Magnetism of CuCl2 ·2D2O and CuCl2 ·2H2O, and of CuBr2 ·6H2O. *J. Magn. Magn. Mater.* **2017**, *434*, 23–29.

(6) Mori, W.; Yamaguchi, K. Spin Glass Behavior of Synthetic Atacamite, Cu2Cl(OH)3. *Mol. Cryst. Liq. Cryst. Sci. Technol. Sect. Mol. Cryst. Liq. Cryst.* **1995**.

(7) Heinze, L.; Beltran-Rodriguez, R.; Bastien, G.; Wolter, A. U. B.; Reehuis, M.; Hoffmann, J.-U.; Rule, K. C.; Süllow, S. The Magnetic Properties of Single-Crystalline Atacamite, Cu2Cl(OH)3. *Phys. B Condens. Matter* **2018**, *536*, 377–378.

(8) Heinze, L.; Jeschke, H. O.; Mazin, I. I.; Metavitsiadis, A.; Reehuis, M.; Feyerherm, R.; Hoffmann, J.-U.; Bartkowiak, M.; Prokhnenko, O.; Wolter, A. U. B.; Ding, X.; Zapf, V. S.; Moya, C. C.; Weickert, F.; Jaime, M.; Rule, K. C.; Menzel, D.; Valentí, R.; Brenig, W.; Süllow, S. Magnetization Process of Atacamite: A Case of Weakly Coupled $S = 1/2$ Sawtooth Chains. *Phys. Rev. Lett.* **2021**, *126* (20), 207201.
